# Body-part specificity for learning of multiple prior distributions in human coincidence timing

**DOI:** 10.1038/s41539-024-00241-x

**Published:** 2024-05-02

**Authors:** Yoshiki Matsumura, Neil W. Roach, James Heron, Makoto Miyazaki

**Affiliations:** 1 Graduate School of Integrated Science and Technology, Shizuoka University, Hamamatsu, Japan; 2 School of Psychology, University of Nottingham, Nottingham, UK; 3 School of Optometry and Vision Science, University of Bradford, Bradford, UK; 4 Faculty of Informatics, Shizuoka University, Hamamatsu, Japan

**Keywords:** Human behaviour, Perception, Sensorimotor processing

## Abstract

During timing tasks, the brain learns the statistical distribution of target intervals and integrates this prior knowledge with sensory inputs to optimise task performance. Daily events can have different temporal statistics (e.g., fastball/slowball in baseball batting), making it important to learn and retain multiple priors. However, the rules governing this process are not yet understood. Here, we demonstrate that the learning of multiple prior distributions in a coincidence timing task is characterised by body-part specificity. In our experiments, two prior distributions (short and long intervals) were imposed on participants. When using only one body part for timing responses, regardless of the priors, participants learned a single prior by generalising over the two distributions. However, when the two priors were assigned to different body parts, participants concurrently learned the two independent priors. Moreover, body-part specific prior acquisition was faster when the priors were assigned to anatomically distant body parts (e.g., hand/foot) than when they were assigned to close body parts (e.g., index/middle fingers). This suggests that the body-part specific learning of priors is organised according to somatotopy.

## Introduction

Sensory signals are inherently variable, but Bayesian estimation^1,2^ can minimise the impact of sensory noise in sensorimotor tasks (e.g., baseball batting in daily tasks). Bayesian estimation involves learning the statistical distribution of a target (e.g., ball speed) and integrating this prior with sensory signals. Psychophysical studies have shown that individuals behave as predicted by the Bayesian estimation model in various sensorimotor tasks such as reaching^1^, force matching^3^, and timing^4,5^. In most previous studies, a single prior distribution was imposed on the participants within certain task sessions. In daily tasks, however, multiple events occur (e.g., fastball and slowball), and each event can have its own unique statistics. Successful Bayesian estimation in real environments relies on the ability to learn multiple prior distributions.

Recently, Roach et al.^6^ demonstrated that when exposed to two different prior distributions (short and long durations) during a timing task, participants first learned a single prior distribution by generalising over the two distributions (‘generalisation’). Then, after approximately 1000 trials, they eventually learned the two independent priors. Moreover, Roach et al. showed that when the two priors were assigned to two different types of motor responses (keypress/vocalisation), participants concurrently learned the two independent priors (‘motor specificity’) within 140 trials. Roach et al. proposed the supplementary motor area (SMA) as a possible neural basis for motor specificity since neurons in the SMA exhibit both time-interval tuning^7^ and action selectivity^8^. A more recent study on monkeys found that neurons in the dorsal frontal regions, including the SMA (pre-SMA and SMA proper), exhibited activity consistent with their Bayesian timing behaviour^9^, supporting the possibility of the proposal.

In the current study, we hypothesised that when two prior distributions are assigned to two different body parts to generate timing responses, participants may concurrently learn two independent prior distributions, even though the type of motor responses is identical (keypress). This hypothesis has a possible neurophysiological basis—the SMA, which is referred to as a possible neural basis of motor specificity^6^, also has somatotopic (body-part specific) activity corresponding to the motor outputs^10,11^. In addition, psychophysical studies suggest that the brain has multiple timers, each of which is associated with different motor effectors (i.e., body parts)^12^. The body-part specific brain structure and/or function may enable individuals to concurrently learn multiple prior distributions. To test the hypothesis for ‘body-part specificity’, we conducted psychophysical experiments using a coincidence timing task.

## Results

**Fig. 1:**
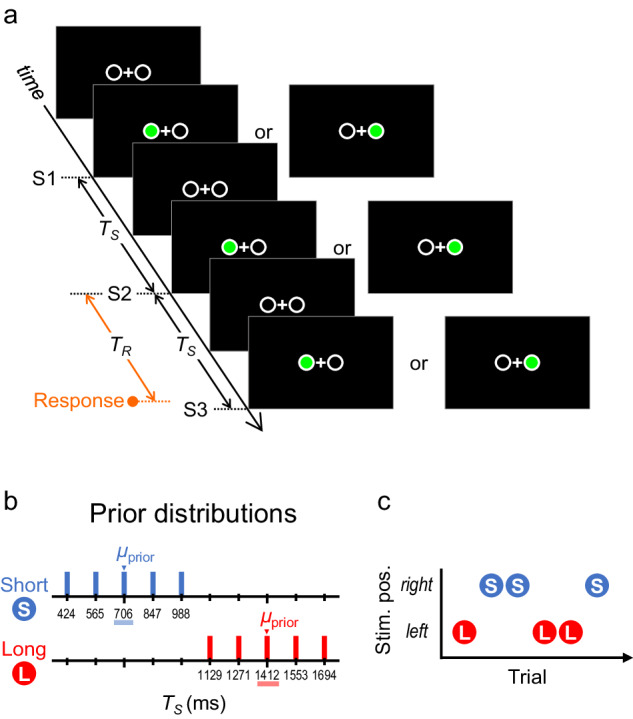
Stimuli and task. **a** Three sequential stimuli (S1 → S2 → S3) were presented on the right or left side of the fixation point. The stimulus time interval (*T*_*S*_) between S1 and S2 and that between S2 and S3 were identical in each trial. Based on the *T*_*S*_ from S1 to S2, participants attempted to press a key to coincide with the onset of S3. The time interval from the onset of S2 to that of the response was measured as the response time interval (*T*_*R*_). **b** *T*_*S*_ was randomly sampled from one of two prior distributions: the short (424, 565, 706, 847, and 988 ms) or long (1129, 1271, 1412, 1553, and 1694 ms) prior. **c** The short and long priors were assigned to the right or left stimuli.

Forty individuals participated in Experiments 1–5 (eight participants per experiment; no overlap among the experiments; for details, see Supplementary Table 1). They performed coincidence timing tasks (Fig. 1a) in which three sequential visual stimuli (S1 → S2 → S3) were presented on the right or left of a fixation point (upper or lower in Exp. 5). The time interval (*T*_*S*_) between S1 and S2 was equal to that between S2 and S3 in each trial. Based on the *T*_*S*_ from S1 to S2, participants attempted to press a key to coincide with the onset of S3. *T*_*S*_ was randomly sampled from a short (424–988 ms; mean [*μ*_prior_] = 706 ms) or long (1129–1694 ms; *μ*_prior_ = 1412 ms) prior distribution (Fig. 1b). Short and long priors were assigned to the right or left stimuli (Fig. 1c) (upper or lower in Exp. 5). Each participant completed 640 trials of the coincidence timing task (320 trials per prior). The time interval between the onset of S2 and response (*T*_*R*_) (Fig. 1a) was used for the analyses.

### Theoretical predictions

**Fig. 2:**
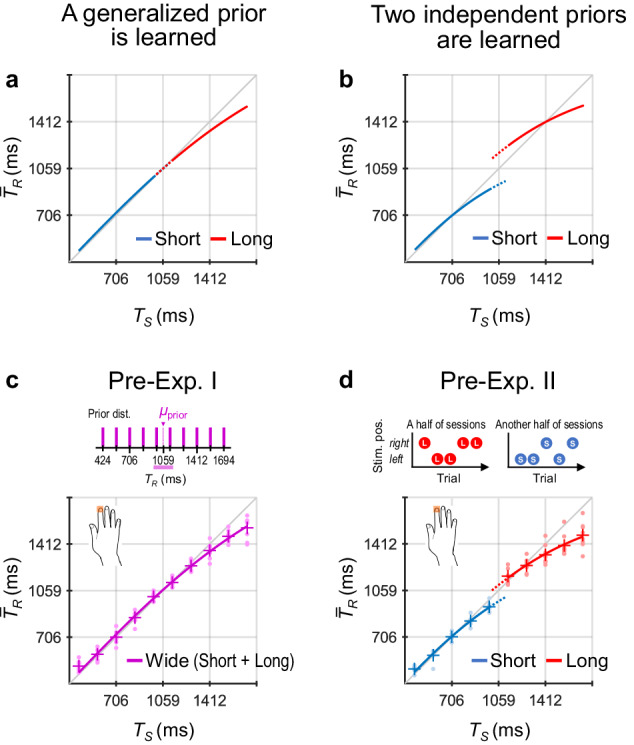
Theoretical predictions. The predictions based on the Bayesian estimation model including the effect of scalar variability (Eq. 4): the mean response time intervals among trials (${\bar{T}}_{R}$) as a function of stimulus time interval (*T*_*S*_) in the case that participants learned a single generalised prior (**a**) and in the case that participants concurrently learned the short and long priors (**b**). In this model, the priors were assumed to have a Gaussian distribution, although we used uniform distributions for the priors in the current experiments (Fig. 1b), based on previous studies^6,15–17,40^. The results of Preliminary Experiments I (**c**) and II (**d**) to verify the theoretical predictions. In Preliminary Experiment I, participants were presented with a single wide prior distribution made by combining the short and long priors. In Preliminary Experiment II, participants were presented with only one of the two priors during half of the sessions, and another prior during the other half. Each graph shows ${\bar{T}}_{R}$ values across participants as a function of *T*_*S*_, which were calculated using data in the last quarter of trials for each prior. The dot markers represent the ${\bar{T}}_{R}$ values of each participant, the plus markers represent the mean ${\bar{T}}_{R}$ values across participants, and the lines represent the curves fitted to the mean ${\bar{T}}_{R}$ values according to the Bayesian estimation model (Eq. 4).

Figure 2a, b shows the theoretical predictions for ${\bar{T}}_{R}$ (mean *T*_*R*_ among trials) as a function of *T*_*S*_. When Bayesian estimation operates, ${\bar{T}}_{R}$ should be biased to the mean of the prior distribution (*μ*_prior_). In addition, including the effect of scalar variability^13,14^ (i.e., greater sensory variability under longer *T*_*S*_), the Bayesian estimation model is expressed as a nonlinear equation (see Eq. 4 in Theoretical Predictions in Methods). According to Eq. 4, the ${\bar{T}}_{R}$ × *T*_*S*_ functions exhibit curves with gentler gradients in the longer *T*_*S*_ because ${\bar{T}}_{R}$ should be biased to the mean of the prior more strongly for longer *T*_*S*_ with greater sensory variability.

This model predicts the experimental results as follows. If participants learned a generalised single prior, the curves for the short and long priors should overlap (Fig. 2a) because ${\bar{T}}_{R}$ should be biased to the single generalised mean of the two priors. In contrast, if participants concurrently learned the short and long priors, two independent curves should appear (Fig. 2b) because ${\bar{T}}_{R}$ should be biased to the respective means of the two priors.

Many earlier studies^5,15–17^ have used the linear models such as Eq. 2 that did not include the effect of scalar variability, which nonetheless well fitted with the experimental results. However, we selected the nonlinear model of Eq. 4 based on the results of preliminary experiments (for details, see Supplementary Methods, Supplementary Results, and Supplementary Fig. 1). In Preliminary Experiment I (Pre-Exp. I, Fig. 2c), participants (*n* = 8) were presented with a single wide prior distribution created by combining the short and long priors. In Preliminary Experiment II (Pre-Exp. II, Fig. 2d), participants (*n* = 8) were presented with the short prior during half of the sessions and with the long prior during the other half. The results were consistent with the theoretical predictions using the nonlinear model of Eq. 4, which was especially evident in Preliminary Experiment I (for details, see Analyses in Supplementary Methods) using a wider range of the prior distribution than those used in previous studies^5,15–17^.

**Fig. 3:**
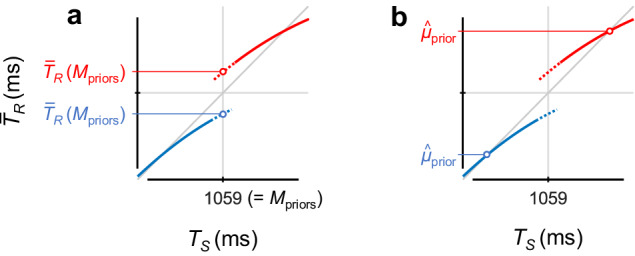
Evaluations of how participants learned the two priors. ${\bar{T}}_{R}({{\rm M}}_{{\rm{priors}}})$: ${\bar{T}}_{R}$ at *T*_*S*_ = 1059 ms (i.e., grand mean over the two priors, *Μ*_priors_) on the fitted curves (**a**). ${\hat{\mu }}_{{\rm{prior}}}$: mean of the acquired prior, which can be inferred from the point that the ${\bar{T}}_{R}$ × *T*_*S*_ curve intersects the unity line (**b**). ${\bar{T}}_{R}({{\rm M}}_{{\rm{priors}}})$ values were calculated for each participant, whereas the ${\hat{\mu }}_{{\rm{prior}}}$ values were calculated using the grand-averaged ${\bar{T}}_{R}$ values (means across participants). The ${\bar{T}}_{R}({{\rm M}}_{{\rm{priors}}})$ values linearly correlated with the ${\hat{\mu }}_{{\rm{prior}}}$ values (see Eq. 5 and Supplementary Fig. 2). If participants learned a single generalised prior, there should be no difference in ${\bar{T}}_{R}({{\rm M}}_{{\rm{priors}}})$ and ${\hat{\mu }}_{{\rm{prior}}}$ between the two priors. However, if participants concurrently learned the two independent priors, ${\bar{T}}_{R}({{\rm M}}_{{\rm{priors}}})$ and ${\hat{\mu }}_{{\rm{prior}}}$ should be greater for the long prior than for the short prior.

To evaluate how participants learned the two priors, we calculated ${\bar{T}}_{R}$ at *T*_*S*_ = 1059 ms (grand mean of the two priors, *Μ*_priors_) on fitted curves [${\bar{T}}_{R}({{\rm M}}_{{\rm{priors}}})$, Fig. 3a]. If participants learned a single generalised prior, there should be no difference in ${\bar{T}}_{R}({{\rm M}}_{{\rm{priors}}})$ between the two priors. However, if participants concurrently learned the two independent priors, then ${\bar{T}}_{R}({{\rm M}}_{{\rm{priors}}})$ should be greater for the long prior than for the short prior.

In theory, the mean of the acquired prior (${\hat{\mu }}_{{\rm{prior}}})$ can be inferred from the point that the ${\bar{T}}_{R}$ × *T*_*S*_ curve intersects the unity line (Fig. 3b). In practice, however, the estimation of this intersection was highly sensitive to variability in individuals’ response patterns (for details, see Analyses in Methods). Therefore, we calculated the ${\hat{\mu }}_{{\rm{prior}}}$ values using the curves fitted to the grand-averaged ${\bar{T}}_{R}$ values (i.e., means across participants), in which the individuals’ idiosyncratic responses were cancelled through averaging. Notably, the ${\hat{\mu }}_{{\rm{prior}}}$ values were accounted for by ${\bar{T}}_{R}({{\rm M}}_{{\rm{priors}}})$ values across participants (*R*^2^ = 0.91, for details, see Eq. 5 and Supplementary Fig. 2).

To facilitate comparisons with earlier studies^15–17^, we also evaluated whether participants concurrently learned the short and long priors using the regression index (1 − slope), which was based on the linear model of Eq. 2. If participants concurrently learned the two priors, the regression indices should be larger than zero (i.e., slope < 1) for both priors, and the index should be greater for the long prior than for the short prior. We verified this using the nonparametric Wilcoxon signed-rank test because the normality for the regression index was rejected (for details, see Analyses in Methods).

### Experiment 1: timing using a single body part (index finger)

**Fig. 4:**
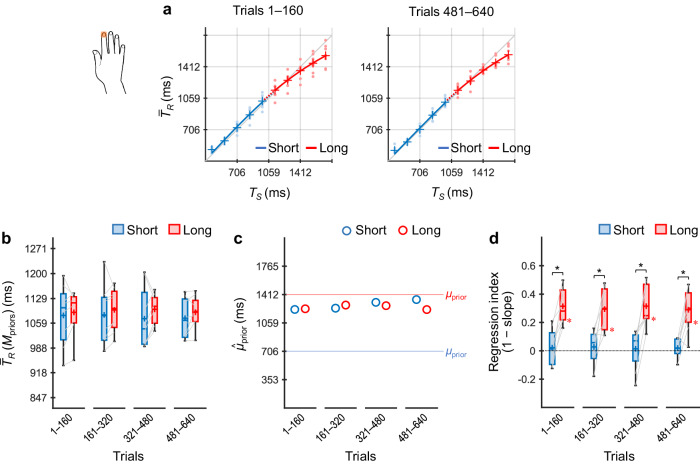
Results of Experiment 1. Participants (*n* = 8) performed the coincidence timing task using only the dominant index finger regardless of the priors. ${\bar{T}}_{R}$ values across participants as a function of *T*_*S*_ for trials 1–160 and 481–640 (**a**). The representations of the markers and lines are the same as those in Fig. 2c, d. ${\bar{T}}_{R}({{\rm M}}_{{\rm{priors}}})$ values across participants (box plots) for the short and long priors, calculated per 160 trials (80 trials/prior) (**b**). The centre line and plus marker in each box represent the median and mean across participants, respectively. The upper and lower limits of each box represent the third and first quartiles (Q3 and Q1), respectively. The upper and lower whiskers indicate the maximum and minimum values within Q3 + 1.5 × IOR (interquartile range) and Q1 – 1.5 × IOR, respectively. The left and right ends of each grey line indicate individual values for the short and long priors, respectively. ${\hat{\mu }}_{{\rm{prior}}}$ values inferred using the grand-averaged ${\bar{T}}_{R}$ values for the short and long priors, calculated per 160 trials (**c**). Regression indices across participants (box plots) for the short and long priors, calculated per 160 trials (**d**), in which the asterisk alongside the lower limit of each box denote that the regression index was significantly greater than zero. * *p*_*cor*_ < 0.05.

Participants (*n* = 8) performed the coincidence timing task using only the dominant index finger, regardless of the stimulus locations (i.e., priors) (Fig. 4).

Figure 4a shows ${\bar{T}}_{R}$ values across participants as a function of *T*_*S*_ for trials 1–160 and trials 481–640 (i.e., trials 1–80 and trials 241–320 per prior). ${\bar{T}}_{R}$ × *T*_*S*_ curves for the short and long priors overlapped in both trial bins. Figure 4b shows ${\bar{T}}_{R}({{\rm M}}_{{\rm{priors}}})$ values across participants in each successive 160 trial bins (80 trial bins/prior). No significant differences between the priors were found (*ps*_*cor*_ ≥ 0.27, *ts*(7) ≤ 1.35, Cohen’s *ds* ≤ 0.48, paired *t*-test with Holm correction), indicating that participants learned a single prior by generalising over the two distributions. This inference is supported by the ${\hat{\mu }}_{{\rm{prior}}}$ values (Fig. 4c), which did not differ systematically between the short and long priors.

Figure 4d shows regression indices across participants, which also did not indicate concurrent learning of the two independent priors. The indices were not significantly greater than zero in all trial bins for the short prior (*ps*_*cor*_ ≥ 0.74, Wilcoxon signed-rank test with Holm correction), although they were significantly greater than zero for the long prior (*ps*_*cor*_ = 0.031), and the difference between the long and short priors remained significant in all trial bins (*ps*_*cor*_ = 0.016).

### Experiment 2: timing using two body parts (index vs middle fingers)

**Fig. 5:**
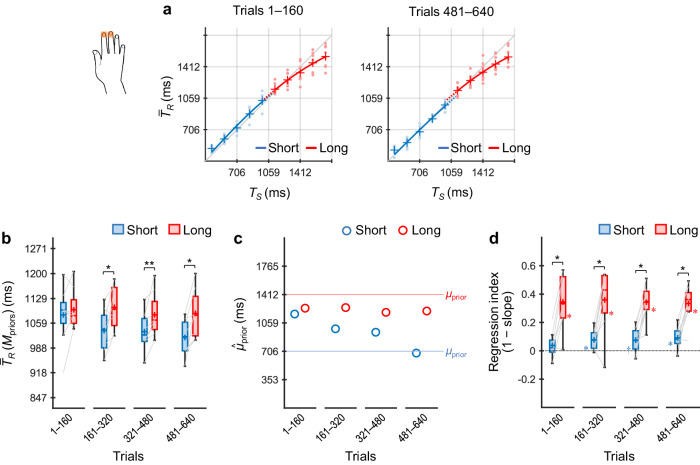
Results of Experiment 2. Participants (*n* = 8) performed the coincidence timing task selectively using the index or middle fingers according to the priors. ${\bar{T}}_{R}$ values across participants as a function of *T*_*S*_ for trials 1–160 and 481–640 (**a**). ${\bar{T}}_{R}({{\rm M}}_{{\rm{priors}}})$ values across participants for the short and long priors (**b**). ${\hat{\mu }}_{{\rm{prior}}}$ values inferred using the grand-averaged ${\bar{T}}_{R}$ values for the short and long priors (**c**). Regression indices across participants for the short and long priors (**d**). The representations of the markers, lines, boxes, and whiskers are the same as those in Fig. 4. ^†^*p* < 0.05, *p_*cor*_ < 0.05, **p_*cor*_ < 0.01.

Next, participants (*n* = 8) performed the coincidence timing task selectively using either the index or middle fingers of the dominant hand according to the stimulus locations (i.e., priors) (Fig. 5).

As shown in Fig. 5a, ${\bar{T}}_{R}$ × *T*_*S*_ curves for the two priors overlapped in trials 1–160 but diverged in trials 481–640. In trials 1–160, ${\bar{T}}_{R}({{\rm M}}_{{\rm{priors}}})$ values showed no difference between the two priors (*p*_*cor*_ = 0.26, *t*(7) = 0.68, *d* = 0.24, paired *t*-test with Holm correction) (Fig. 5b). In trials 161–320 and later, however, the ${\bar{T}}_{R}({{\rm M}}_{{\rm{priors}}})$ values were significantly greater for the long prior than for the short prior (*ps*_*cor*_ ≤ 0.025, *ts*(7) ≥ 2.84, *ds* ≥ 1.00). A similar profile was also evident in the ${\hat{\mu }}_{{\rm{prior}}}$ values (Fig. 5c), indicating that participants acquired a single generalised prior in the early trials and subsequently learned the two independent priors.

Figure 5d shows regression indices across participants. For the short prior, the regression index did not differ significantly from zero in trials 1–160 (*p*_*cor*_ = 0.13, Wilcoxon signed-rank test with Holm correction) but did in trials 161–320 and 481–640 (*ps*_*cor*_ ≤ 0.039). In trials 321–480, the index was greater than zero without the correction (*p* = 0.027) although the difference did not reach significance with the correction (*p*_*cor*_ = 0.055). The indices for the long prior were significantly greater than zero (*ps*_*cor*_ ≤ 0.039), and the difference between the long and short priors remained significant in all trial bins (*ps*_*cor*_ ≤ 0.023). These results were generally consistent with those of the ${\bar{T}}_{R}({{\rm M}}_{{\rm{priors}}})$ values.

### Experiment 3: timing using two body parts (right vs left hands)

**Fig. 6:**
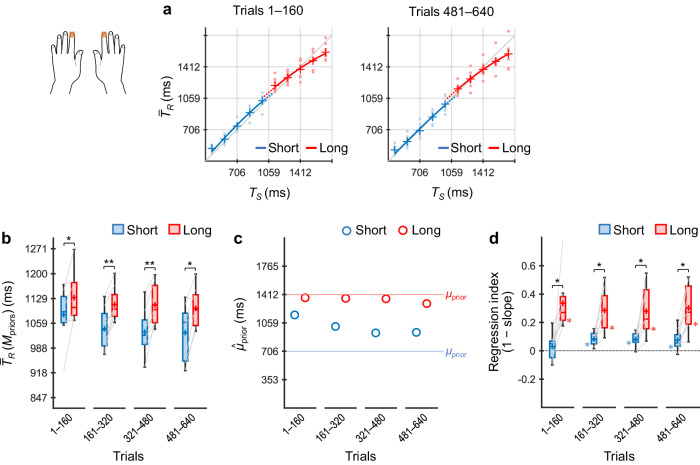
Results of Experiment 3. Participants (*n* = 8) performed the coincidence timing task selectively using the right or left index finger according to the priors. ${\bar{T}}_{R}$ values across participants as a function of *T*_*S*_ for trials 1–160 and 481–640 (**a**). ${\bar{T}}_{R}({{\rm M}}_{{\rm{priors}}})$ values across participants for the short and long priors (**b**). ${\hat{\mu }}_{{\rm{prior}}}$ values inferred using the grand-averaged ${\bar{T}}_{R}$ values for the short and long priors (**c**). Regression indices across participants for the short and long priors (**d**). The representations of the markers, lines, boxes, and whiskers are the same as those in Fig. 4. *p_*cor*_ < 0.05, **p_*cor*_ < 0.01.

Participants (*n* = 8) next performed the coincidence timing task selectively using their right or left index fingers according to the stimulus locations (i.e., priors) (Fig. 6).

In this instance, ${\bar{T}}_{R}$ × *T*_*S*_ curves diverged between the two priors in both trials 1–160 and 481–640 (Fig. 6a), and ${\bar{T}}_{R}({{\rm M}}_{{\rm{priors}}})$ values across participants were significantly greater for the long prior than for the short prior in all trial bins (*ps*_*cor*_ ≤ 0.026, *ts*(7) ≥ 2.33, *ds* ≥ 0.82, paired *t*-test with Holm correction) (Fig. 6b). The ${\hat{\mu }}_{{\rm{prior}}}$ values show similar profiles to the ${\bar{T}}_{R}({{\rm M}}_{{\rm{priors}}})$ values (Fig. 6c). These results suggest that the participants concurrently learned the two independent priors from the early trials.

As shown in Fig. 6d, the regression index for the short prior exhibited no significant difference from zero in trials 1–160 (*p*_*cor*_ = 0.23, Wilcoxon signed-rank test with Holm correction), although the indices were significantly greater than zero in trials 161–320 and later (*ps*_*cor*_ ≤ 0.031). The indices for the long prior were significantly greater than zero in all trial bins (*ps*_*cor*_ = 0.031) and greater than those for the short prior in all trial bins (*ps*_*cor*_ = 0.016).

Thus, the regression indices did not indicate central tendency for the short prior in trials 1–160, although did for the long prior. This did not support the concurrent learning of the two independent priors in the early trials, which was not consistent with the results of the ${\bar{T}}_{R}({{\rm M}}_{{\rm{priors}}})$ values. Notably, the ${\hat{\mu }}_{{\rm{prior}}}$ value for the short prior in trials 1–160 was 1158.1 ms (Supplementary Table 2), which was greater than the short prior (424–988 ms) and was included in the long prior (1129–1694 ms). According to the Bayesian estimation model, ${\bar{T}}_{R}$ should be biased to 1158.1 ms. In this case, the regression index should be 0 (i.e., slope = 1), which is computed from the ${\bar{T}}_{R}$ values as a function of *T*_*S*_ with substituting 1158.1 ms, 365.3 ms (Supplementary Table 3), and 0.194 (Supplementary Table 4) (or 1158.1 ms, 256.9 ms [Supplementary Table 5], and 0.136 [Supplementary Table 6]) into *μ*_prior,_ *σ*_prior_, and *w* in Eq. 4, respectively. This was consistent with the experiment result. Thus, Bayesian estimation should not appear as the ‘central’ tendency when ${\hat{\mu }}_{{\rm{prior}}}$ was largely biased towards the other prior.

### Experiment 4: timing using two body parts (contralateral hand vs foot)

**Fig. 7:**
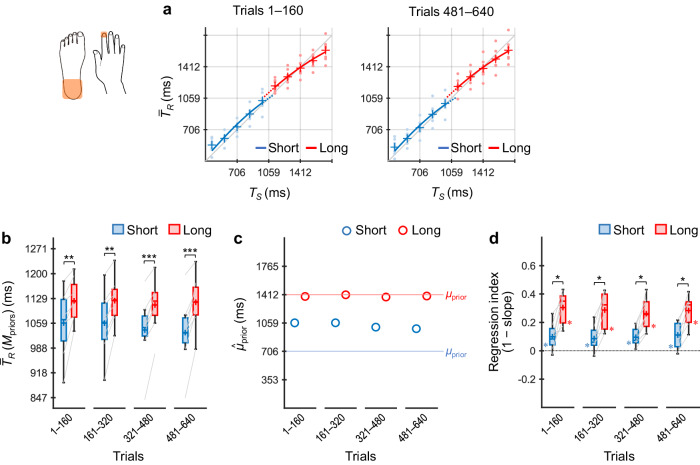
Results of Experiment 4. Participants (*n* = 8) performed the coincidence timing task selectively using the contralateral hand or foot according to the priors. ${\bar{T}}_{R}$ values across participants as a function of *T*_*S*_ for trials 1–160 and 481–640 (**a**). ${\bar{T}}_{R}({{\rm M}}_{{\rm{priors}}})$ values across participants for the short and long priors (**b**). ${\hat{\mu }}_{{\rm{prior}}}$ values inferred using the grand-averaged ${\bar{T}}_{R}$ values for the short and long priors (**c**). Regression indices across participants for the short and long priors (**d**). The representations of the markers, lines, boxes, and whiskers are the same as those in Fig. 4. *p_*cor*_ < 0.05, **p_*cor*_ < 0.01, ***p_*cor*_ < .001.

Participants (*n* = 8) performed the coincidence timing task selectively using their right/left index finger or left/right heel according to the stimulus locations (i.e., priors) (Fig. 7).

${\bar{T}}_{R}$ × *T*_*S*_ curves for the two priors diverged in both trials 1–160 and 481–640 (Fig. 7a). ${\bar{T}}_{R}({{\rm M}}_{{\rm{priors}}})$ values were significantly greater for the long prior than for the short prior over all trial bins (*ps*_*cor*_ ≤ 0.0026, *ts*(7) ≥ 3.99, *ds* ≥ 1.41, paired *t*-test with Holm correction) (Fig. 7b). Although the normality for the residuals was not rejected for the ${\bar{T}}_{R}({{\rm M}}_{{\rm{priors}}})$ values, it was marginal (*p* = 0.077) in Experiment 4 (for details, see Analyses in Methods). Therefore, we supplementarily conducted Wilcoxon signed-rank tests with Holm correction on the ${\bar{T}}_{R}({{\rm M}}_{{\rm{priors}}})$ values, which also showed that the ${\bar{T}}_{R}({{\rm M}}_{{\rm{priors}}})$ values were significantly greater for the long prior than for the short prior over all trial bins (*ps*_*cor*_ ≤ 0.021). The ${\hat{\mu }}_{{\rm{prior}}}$ values show a similar profile to the ${\bar{T}}_{R}({{\rm M}}_{{\rm{priors}}})$ values (Fig. 7 c). The results indicate that the participants learned the two independent priors from the early trials.

As shown in Fig. 7d, regression indices were significantly greater than zero for the short and long priors in all trial bins (*ps*_*cor*_ ≤ 0.031, Wilcoxon signed-rank test with Holm correction). The indices were greater for the long prior than for the short prior in all trial bins (*ps*_*cor*_ = 0.016). The results were consistent with those of the ${\bar{T}}_{R}({{\rm M}}_{{\rm{priors}}})$ values.

### Experiment 5: timing using two body parts (ipsilateral hand vs foot)

**Fig. 8:**
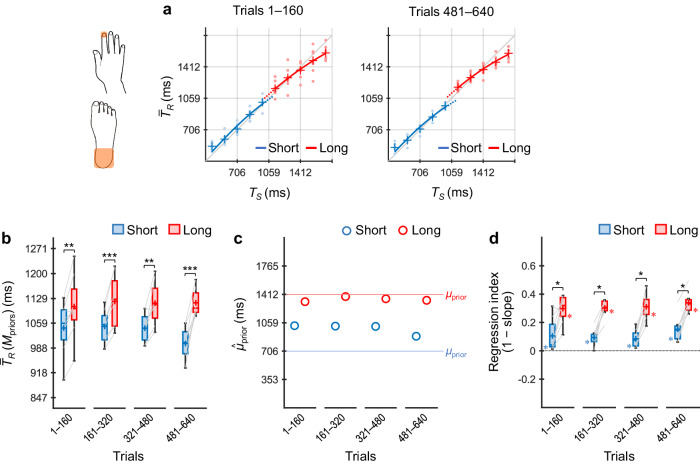
Results of Experiment 5. Participants (*n* = 8) performed the coincidence timing task selectively using the ipsilateral hand or foot according to the priors. ${\bar{T}}_{R}$ values across participants as a function of *T*_*S*_ for trials 1–160 and 481–640 (**a**). ${\bar{T}}_{R}({{\rm M}}_{{\rm{priors}}})$ values across participants for the short and long priors (**b**). ${\hat{\mu }}_{{\rm{prior}}}$ values inferred using the grand-averaged ${\bar{T}}_{R}$ values for the short and long priors (**c**). Regression indices across participants for the short and long priors (**d**). The representations of the markers, lines, boxes, and whiskers are the same as those in Fig. 4. *p_*cor*_ < 0.05, **p_*cor*_ < 0.01, ***p_*cor*_ < 0.001.

Participants (*n* = 8) performed the coincidence timing task selectively using their dominant index finger or the ipsilateral heel according to the stimulus locations (i.e., priors) (Fig. 8). To maintain stimulus–response compatibility, stimuli were presented above or below the fixation point. The participants pressed a key with their index finger when the stimuli were presented above, and they pressed a foot key with their heel when the stimuli were presented below.

${\bar{T}}_{R}$ × *T*_*S*_ curves for the two priors diverged in both trials 1–160 and 481–640 (Fig. 8a), with ${\bar{T}}_{R}({{\rm M}}_{{\rm{priors}}})$ values remaining significantly greater for the long prior than for the short prior over all trial bins (*ps*_*cor*_ ≤ 0.0035, *ts*(7) ≥ 3.94, *ds* ≥ 1.39, paired *t*-test with Holm correction) (Fig. 8b). The ${\hat{\mu }}_{{\rm{prior}}}$ values showed a similar profile to the ${\bar{T}}_{R}({{\rm M}}_{{\rm{priors}}})$ values (Fig. 8c).

Regression indices were significantly greater than zero for the short and long priors in all trial bins (*ps*_*cor*_ = 0.031, Wilcoxon signed-rank test with Holm correction) (Fig. 8d). The indices were greater for the long prior than for the short prior in all trial bins (*ps*_*cor*_ ≤ 0.016). The results were consistent with those of the ${\bar{T}}_{R}({{\rm M}}_{{\rm{priors}}})$ values.

These results indicate that participants could quickly learn two independent priors when they were associated with body parts located on separate limbs, even when the limbs did not span the right and left body sides (as was the case in Experiment 4).

### Comparisons across the experiments

In the results of each experiment, we tested whether participants concurrently learned the short and long priors over time. To compare ${\bar{T}}_{R}({{\rm M}}_{{\rm{priors}}})$ values across experiments, we conducted a three-way analysis of variance (ANOVA) with one between-participant factor (5 experiments) and two within-participant factors (2 priors × 4 trial bins). Significant main effects were found for prior (*p* < 0.001, *F*(1, 35) = 98.73, *η*_*p*_^2^ = 0.74) and trial bin (*p* = 0.020, *F*(1.69, 59.17) = 4.50, *η*_*p*_^2^ = 0.11), although the main effect of experiment was not significant (*p* = 0.98, *F*(4, 35) = 0.12, *η*_*p*_^2^ = 0.013). Significant two-way interactions were found between experiment and prior (*p* = 0.011, *F*(4, 35) = 3.83, *η*_*p*_^2^ = 0.30) and between prior and trial bin (*p* < 0.001, *F*(3, 105) = 5.98, *η*_*p*_^2^ = 0.15). However, the interaction between experiment and trial bin (*p* = 0.63, *F*(6.76, 59.17) = 0.74, *η*_*p*_^2^ = 0.078) and that among the three factors (*p* = 0.33, *F*(12, 105) = 1.14, *η*_*p*_^2^ = .12) were not significant.

The analyses of simple effects for the interaction between experiment and prior revealed that the effect of prior was non-significant in Experiment 1 (*p* = 0.20, *F*(1, 7) = 1.98, *η*_*p*_^2^ = 0.22) but was significant in Experiments 2–5 (*ps* ≤ 0.0095, *Fs*(1, 7) ≥ 12.50, *η*_*p*_^2^*s* ≥ 0.64). The results further supported that a single generalised prior was learned in Experiment 1 (Fig. 4), whereas the two independent priors were learned in Experiments 2–5 (Figs. 5–8).

The analyses of simple effects for the interaction between prior and trial bin revealed that the effect of trial bin was not significant for the long prior (*p* = 0.46, *F*(2.16, 75.54) = 0.81, *η*_*p*_^2^ = 0.023), but it was significant for the short prior (*p* = 0.0017, *F*(1.85, 64.74) = 7.35, *η*_*p*_^2^ = 0.17). Consistent with the statistical results, ${\bar{T}}_{R}({{\rm M}}_{{\rm{priors}}})$ and ${\hat{\mu }}_{{\rm{prior}}}$ values for the long prior showed little change among trial bins in all experiments, but those for the short prior generally decreased over trials in Experiments 2–5. These results suggest that, when the two independent priors were learned, the acquisition of the long prior was completed in the early trials, whereas that of the short prior was developed as the trials progressed.

## Discussion

The current results supported our hypothesis of body-part specificity. When participants used only one body part for timing responses, they learned a single prior by generalising over two prior distributions (Exp. 1). However, when participants selectively used two body parts, they concurrently learned the two independent priors (Exp. 2–5). Moreover, the results revealed that body-part specific learning of the priors was more quicky attained when the priors were assigned to anatomically distant body parts (e.g., hand vs foot) than when they were assigned to closer body parts (e.g., index vs middle finger). Thus, body-part specific learning of the priors was regulated in a somatotopic manner.

We hypothesised body-part specificity because the SMA, which was proposed as a possible neural basis for motor specificity^6^, has somatotopy^10,11^. The current results supported the hypothesis and exhibited further consistency with the neurophysiological properties of the SMA. In the SMA, there exist neurons that are activated specifically by movement of the contralateral hand^10,18^. The hand and leg movements are mapped in the relatively rostral and caudal regions in the SMA, respectively^11^. Thus, the body parts that are anatomically distant (or close) are also represented in the anatomically distant (or close) regions in the SMA. Accordingly, we inferred the following somatotopic organisation of body-part associated learning of the priors: when the neural representations of the body parts in the SMA are identical and close, the assigned priors are generalised. However, when those in the SMA are distant, the assigned priors are independently acquired.

In addition, the cerebellum and basal ganglia (BG) should be also referred as the possible neural bases for body-part specificity. The cerebellum is involved in precise timing^19^ and has somatotopy^20^. The neural circuit model of the cerebellum can learn and represent the prior distribution of time intervals^21^, and the outputs of the circuit replicate the psychophysical observations^4,5^. The BG is a core neural basis that encodes time intervals and exhibits neuronal responses that reflect scalar variability^13^. The BG also exhibits somatotopy^22^. Moreover, a neuroimaging study suggested that BG represents prior uncertainty in a visuospatial decision-making task^23^. Notably, the SMA has neuronal connectivity with the cerebellum and BG^24^. Therefore, these possible neural bases are not always mutually exclusive. The SMA, cerebellum, and BG may constitute a neuronal network to generate Bayesian estimation that can learn multiple priors in timing. However, the abovementioned discussion is merely speculative. Future neuroimaging or neurophysiological experiments are necessary to identify the actual neural bases of body-part specificity.

The body-part specificity found in the present study does not necessarily imply that prior distributions are represented or implemented within motor processing. Psychophysical^17^ and psychophysiological^25^ studies indicated that the prior distribution affects the perceptual process during timing tasks. There is growing evidence that motor responses or programs affect various types of time perception^26–30^. Thus, it is also plausible that motor responses affect the Bayesian learning of perceptual timing. Notably, a meta-analysis of neuroimaging studies showed that the SMA was consistently activated across various motor and perceptual timing tasks^31^. We propose the SMA as one of the possible neural bases of body-part specificity. If it is true, the present and previous results are consistent with each other.

We can also infer functional significance in motor-perception coupling in the Bayesian learning of timing. During sensorimotor tasks, the variety of motor responses is generally greater when using multiple body parts than when using only a single body part. It should be rational to increase the variety of the perceptual strategy according to the increase in the variety of the motor responses. Inversely, when the variety of the motor response is limited, it would be reasonable for efficient utilisation of a finite perceptual resource to narrow down variety in the perceptual strategy.

An unexpected feature of our results was that when participants learned the two independent priors, they quickly completed the acquisition of the long prior but needed more trials to learn the short prior. Similar results were not found in Preliminary Experiment II (see Supplementary Results and Supplementary Fig. 1c, d). Therefore, such preferential acquisition of the long prior occurred when the short and long priors were intermixed within a session. A similar effect was also found by ref. ^6^. When participants eventually learned the two priors after excessive trials, it was attained by shifting the ${\hat{\mu }}_{{\rm{prior}}}$ for the short prior away from that for the generalised prior (Fig. 4 in Roach et al.). Although the actual mechanism for the preferential acquisition of the long prior is unclear at this stage, it may be related to sensory uncertainty. Due to scalar variability^13^^,^^14^, there is greater sensory uncertainty in longer stimulus intervals. According to the Bayesian estimation model, greater sensory uncertainty results in stronger dependence of time estimation on the prior to compensate for the uncertainty. Faster acquisition for the long prior may reflect a prioritisation of learning towards higher sensory uncertainty contexts where additional prior knowledge will be more impactful.

In the current study, we focused on the means of the prior distributions acquired in the brain (${\hat{\mu }}_{{\rm{prior}}}$) to evaluate whether participants learned the two independent priors. It remains unclear as to how participants learn the variability (*σ*_prior_) of the prior distributions. The *σ*_prior_ acquired in the brain (${\hat{\sigma }}_{{\rm{prior}}}$) and Weber fraction (*w*) are unspecified by the fitted curves (*cf*. Supplementary Tables 3–6). Therefore, we cannot directly discuss the ${\hat{\sigma }}_{{\rm{prior}}}$ values. Meanwhile, the ${\hat{\mu }}_{{\rm{prior}}}$ values linearly correlated with the ${\bar{T}}_{R}({{\rm M}}_{{\rm{priors}}})$ values across the experiments (Supplementary Fig. 2), on which the values for the wide prior (Pre-Exp. I, purple crosses) were plotted with little deviation. This suggests that there was little difference in the ${\hat{\sigma }}_{{\rm{prior}}}$ and *w* values between Preliminary Experiment I and other experiments (for details, see Eq. 5), although the wide prior had a greater *σ*_prior_ than the short and long priors. It was suggested that the learning of *μ*_prior_ is attained by a relatively smaller number of trials^32^, whereas that of *σ*_prior_ needs a greater number of trials^5^. The participants might first acquire a generalised wide ${\hat{\sigma }}_{{\rm{prior}}}$ and not be able to fully learn the two independent narrower ${\hat{\sigma }}_{{\rm{prior}}}$ within 640 trials, even when they learned the ${\hat{\mu }}_{{\rm{prior}}}$ of the two independent priors. Previous studies also measured the *w* values by using time-interval judgement tasks^15,16^. This methodology enables to directly evaluate both ${\hat{\mu }}_{{\rm{prior}}}$ and ${\hat{\sigma }}_{{\rm{prior}}}$ values in future studies.

Whether body-part specificity also operates in nontemporal tasks remains unclear as well. Notably, as a ‘side effect’, the association between the body parts and prior distributions was found in the judgement of visual motion directions^33^, although the causal relationship between the motor responses and priors was opposite to that in our tasks. In this study, participants triggered the motion stimulus (upward/downward) by key pressing of the right or left index finger. Then, they judged the motion direction. The right and left keypresses were associated with either of two prior distributions (upward/downward). Consequently, their judgements were biased to the means of the priors. The ecological validity of this effect was unclear, since such a causality from motor responses to visual statistics is not easily found in daily environments. However, this finding suggests that the brain is capable of associating the body parts with priors in the nontemporal task. In addition, in a spatial aiming task, dependency on a prior differed according to whether a typical reaching or atypical wrist-rotation setting was used^34^. Based on the result, the authors proposed that a single prior could be learned differently across different effectors (finger/wrist). This mechanism may also enable to learn multiple priors in the spatial task.

Moreover, it will be instructive to investigate daily human behaviours such as sports^35–38^. Skilful baseball or cricket players may utilise body-part specificity. For example, to improve the hit rates, a batter may beat time using a foot when a fastball is pitched (i.e., short prior), whereas using a hand when a slowball is pitched (i.e., long prior). In addition, future studies on people with autism can further the current findings. Previous studies demonstrated that individuals with autism or high autistic traits have a disability in learning or utilising the prior distribution in time processing^16,39^. People with autism may also exhibit their specific behaviour in the learning or utilisation of the multiple prior distributions. Our finding would enhance the application of the Bayesian approach to our daily behaviour.

## Methods

### Participants

Forty healthy individuals participated in Experiments 1–5. In addition, 16 healthy individuals participated in Preliminary Experiments I and II (see Supplementary Methods). Eight individuals participated in one of seven experiments (for the profiles of participants in each experiment, see Supplementary Table 1). There was no overlap of participants among the experiments to avoid a possible effect of the priors learned in the previous experiment. All participants were naïve to the purpose of the experiments.

This study was approved by the Ethics Committee of Shizuoka University (15–19). All experiments were performed in accordance with the approved guidelines and regulations. All participants provided written informed consent.

### Stimuli

In a dimly lit sound-shielded room, each participant placed their head on a chin rest and sat at the distance of 87 cm from the monitor (Sony GDM-F520, Japan; 85 Hz). Presentation software (Neurobehavioral Systems, USA) was used for generating the stimuli and recording responses of the participants.

Three sequential stimuli (S1 → S2 → S3) were presented on the right or left side of the fixation point (Fig. 1a) in Experiments 1–4 or the upper or lower side in Experiment 5. The duration of each stimulus was 106 ms. The diameter of the frame circles in which stimuli (green emission) appeared was 1.1° in visual angle, and the distance between the centre of the two circles was 2.2°. The stimulus time interval (*T*_*S*_) between S1 and S2 and that between S2 and S3 were identical within a trial.

For each trial, *T*_*S*_ was randomly sampled from either of two discrete uniform prior distributions (Fig. 1b): the short prior (424, 565, 706, 847, and 988 ms; *μ*_prior_ = 706 ms) or the long prior (1129, 1271, 1412, 1553, and 1694 ms; *μ*_prior_ = 1412 ms). The short and long priors were assigned to the right or left stimuli (Fig. 1c) in Experiments 1–4 or the upper or lower stimuli in Experiment 5. The combinations between the priors (short/long) and stimulus locations (right/left or upper/lower) were counterbalanced among participants in each experiment. The trial-by-trial order of the priors (i.e., stimulus locations) was randomly determined with the restriction that *T*_*S*_ was not repetitively sampled from the same prior for more than four trials.

### Task

Based on *T*_*S*_ from S1 to S2, participants attempted to press a key to coincide with the onset of S3 (coincidence timing task). In this task, there was no additional signal to feedback the accuracy of the response timing to the participants. Participants were instructed not to move their gaze but to fixate the fixation point during the task. For each trial, the time interval from the onset of S2 to that of the motor response was measured as the response time interval (*T*_*R*_) (Fig. 1a). The key pressing was conducted using the dominant index finger only (Exp. 1), dominant index or middle finger (Exp. 2), right or left index finger (Exp. 3), right/left index finger or left/right heel (Exp. 4), or dominant index finger or ipsilateral heel (Exp. 5). In Experiments 2–5, the two body parts that responded were assigned to the compatible stimulus locations (e.g., responding by the right [left] hand to the right [left] stimuli). According to the combinations between the priors and stimulus locations, those between the priors and body parts were counterbalanced among participants in Experiments 2–5. A single key was used in Experiment 1. Two keys were used in Experiments 2–5. The horizontal distance between the centres of the right and left keys was 1.9 cm in Experiment 2, 11.5 cm in Experiment 3, and that between the contralateral hand and foot keys was 11.5 cm in Experiment 4. The centres of the hand and foot keys were approximately placed at the same horizontal position in Experiment 5.

### Procedure

Each participant completed 640 trials (40 trials/session × 16 sessions) of the task. The interval from the onset of S3 in a trial to that of S1 in the subsequent trial was 3.1 s. A short beep (0.2 s) was presented 1 s before S1 to alert participants to the beginning of the trial. Participants took a 1-min break after each session and a 5-min break per 4 sessions. When participants reported fatigue or drowsiness, the break was extended.

### Theoretical predictions

According to the Bayesian estimation theory^1,2^, the brain integrates the sensory input of a target (*X*_sensed_) and the prior distribution about the target to obtain the optimal estimate of the target (*X*_estimated_), as follows:

1 $${X}_{\text{estimated}}=\frac{{\sigma }_{\text{prior}}^{2}}{{\sigma }_{\text{prior}}^{2}+{\sigma }_{\text{sensed}}^{2}}{X}_{\text{sensed}}+\frac{{\sigma }_{\text{sensed}}^{2}}{{\sigma }_{\text{prior}}^{2}+{\sigma }_{\text{sensed}}^{2}}{\mu }_{\text{prior}}$$

where *σ*_sensed_ denotes the standard deviation of *X*_sensed_ (i.e., degree of sensory variability). *μ*_prior_ and *σ*_prior_ denote the mean and standard deviation of the prior distribution, respectively.

Assuming that there is no bias at the stages of sensory inputs and motor outputs in the coincidence timing task, *X*_sensed_ can be approximated by *T*_*S*_, and the mean among trials of *X*_estimated_ can be approximated by that of *T*_*R*_ (${\bar{T}}_{R})$. Accordingly, ${\bar{T}}_{R}$ can be expressed as a linear function of *T*_*S*_ as follows:

2 $${\bar{T}}_{\text{R}}=\frac{{\sigma }_{\text{prior}}^{2}}{{\sigma }_{\text{prior}}^{2}+{\sigma }_{\text{sensed}}^{2}}{T}_{\text{S}}+\frac{{\sigma }_{\text{sensed}}^{2}}{{\sigma }_{\text{prior}}^{2}+{\sigma }_{\text{sensed}}^{2}}{\mu }_{\text{prior}}$$

where we assumed the prior distribution as a Gaussian distribution, although the uniform distributions were used for the priors, based on previous studies^6,15–17,40^. In Eq. 2, $\frac{{\sigma }_{\text{prior}}^{2}}{{\sigma }_{\text{prior}}^{2}+{\sigma }_{\text{sensed}}^{2}}$ represents the slope of ${\bar{T}}_{R}$ against *T*_*S*_, and $\frac{{\sigma }_{\text{sensed}}^{2}}{{\sigma }_{\text{prior}}^{2}+{\sigma }_{\text{sensed}}^{2}}{\mu }_{\text{prior}}$ represents the intercept. When participants learned a prior distribution, the slope should be smaller than 1 (‘central tendency’). The central tendency reflects that the estimate of *T*_*S*_ is biased to *μ*_prior,_ due to Bayesian estimation. The greater central tendencies appear as the smaller slopes.

In addition, according to scalar variability^13,14^, *σ*_sensed_ should be scaled per *T*_*S*_ as follows:

3 $${\sigma }_{\text{sensed}}=w{T}_{S}$$

where *w* denotes the Weber fraction. Then, Eq. 2 can be transformed as

4 $${\bar{T}}_{\text{R}}=\frac{{\sigma }_{\text{prior}}^{2}}{{\sigma }_{\text{prior}}^{2}+{w}^{2}{{T}_{\text{S}}}^{2}}{T}_{\text{S}}+\frac{{w}^{2}{{T}_{\text{S}}}^{2}}{{\sigma }_{\text{prior}}^{2}+{w}^{2}{{T}_{\text{S}}}^{2}}{\mu }_{\text{prior}}$$

Equation 4 predicts that the ${\bar{T}}_{R}$ × *T*_*S*_ functions exhibit nonlinear profile with a gentler gradient in the longer *T*_*S*_ (Fig. 2a, b).

Figure 2a shows the prediction when participants learned a single generalised prior. Figure 2b shows the prediction when participants concurrently learned the short and long priors. As shown in Fig. 2c, d, these predictions were supported by the results of Preliminary Experiments I and II (for details, see Supplementary Methods, Supplementary Results, and Supplementary Fig. 1).

### Analyses

We measured *T*_*R*_ in each trial (Fig. 1a). We excluded trials containing any of the following responses from analyses: no key pressing, pressing the opposite key, pressing the key twice or more. The rates of the excluded responses were 0.38% over all experiments (Exp. 1: 0.08%, Exp. 2: 0.45%, Exp. 3: 0.51%, Exp. 4: 0.72%, Exp. 5: 0.64%, Pre-Exp. I: 0.08%, Pre-Exp. II: 0.18%). We sorted the *T*_*R*_ values for each *T*_*S*_ every 160 trials (80 trials per prior) to calculate the ${\bar{T}}_{R}$ values for each prior. Then, we fitted the ${\bar{T}}_{R}$ values as a function of *T*_*S*_ by Eq. 4 using the least squares method (Matlab R2022b with the Optimisation Toolbox).

As shown in Fig. 3a, we calculated ${\bar{T}}_{R}$ at *T*_*S*_ = 1059 ms (i.e., grand mean of the short and long priors, *Μ*_priors_) on the fitted curves [${\bar{T}}_{R}({{\rm M}}_{{\rm{priors}}})$]. The ${\bar{T}}_{R}({{\rm M}}_{{\rm{priors}}})$ values should have no difference between the short and long priors if participants learned a single prior by generalising over the two distributions. Meanwhile, the ${\bar{T}}_{R}({{\rm M}}_{{\rm{priors}}})$ values should be greater for the long prior than for the short prior if participants concurrently learned the two independent priors.

We calculated the ${\bar{T}}_{R}({{\rm M}}_{{\rm{priors}}})$ values for each prior per trial bin in each participant. The normality of the residuals of the ${\bar{T}}_{R}({{\rm M}}_{{\rm{priors}}})$ values was not rejected over Experiments 1–5 (*p* = 0.15, Shapiro–Wilk normality test) and in each experiment (Exp. 1: *p* = 0.50, Exp. 2: *p* = 0.35, Exp. 3: *p* = 0.73, Exp. 4: *p* = 0.077, Exp. 5: *p* = 0.54). We tested whether the ${\bar{T}}_{R}({{\rm M}}_{{\rm{priors}}})$ values were greater for the long prior than for the short prior in each experiment, using one-tailed paired *t*-tests corrected by the Holm method (corrected *p*-values [*p*_*cor*_] are shown). We used Cohen’s *d*^41^ for the size effect index of the *t*-test. Regarding Experiment 4, the *p*-value for the normality test was greater than 0.05 but less than 0.1. Therefore, we supplementarily conducted one-tailed Wilcoxon signed-rank tests on the ${\bar{T}}_{R}({{\rm M}}_{{\rm{priors}}})$ values in Experiment 4 for strict evaluation.

Moreover, for the comparisons across the experiments, we conducted a three-way ANOVA with one between-participant factor (experiment) and two within-participant factors (prior, trial bin) on the ${\bar{T}}_{R}({{\rm M}}_{{\rm{priors}}})$ values. In the ANOVA, the equality of variance for trial bin was rejected by Mendoza’s multisample sphericity test (*p* < 0.001); therefore, we adjusted the relevant degrees of freedom using Greenhouse–Geisser’s *ε*. We used *η*_*p*_^2^ for the size effect index of the ANOVA. The ANOVA and subsequent analyses were carried out using R version 4.2.2 and the R function ‘anovakun’ version 4.8.7.

In theory, the mean of the acquired prior (${\hat{\mu }}_{{\rm{prior}}}$) can be inferred from the point that the ${\bar{T}}_{R}$ × *T*_*S*_ curve intersects the unity line (i.e., the point of ${\bar{T}}_{R}$ = *T*_*S*_) (Fig. 3b). Substituting ${\bar{T}}_{R}$ into *T*_*S*_ (or *T*_*S*_ into ${\bar{T}}_{R}$), Eq. 4 derives ${\bar{T}}_{R}$ = *μ*_prior_ (or *T*_*S*_ = *μ*_prior_). The *μ*_prior_ estimated from the participant’s responses should reflect the ${\hat{\mu }}_{{\rm{prior}}}$. However, the estimation of the intersection is highly sensitive to idiosyncratic responses (e.g., overshoots, undershoots, steeper slopes than unity), leading to implausible ${\hat{\mu }}_{{\rm{prior}}}$ values (e.g., <0 ms, >2000 ms) being obtained in 14.4% of the curve fittings. Therefore, we could not use the ${\hat{\mu }}_{{\rm{prior}}}$ values for the statistical tests.

Instead, we calculated the ${\hat{\mu }}_{{\rm{prior}}}$ values using the grand-averaged ${\bar{T}}_{R}$ values (mean across participants). There was no implausible value when inferring ${\hat{\mu }}_{{\rm{prior}}}$ using the curves fitted to the grand-averaged ${\bar{T}}_{R}$ values because idiosyncratic responses for each participant were cancelled by averaging across participants. The ${\hat{\mu }}_{{\rm{prior}}}$ values linearly correlated with the ${\bar{T}}_{R}({{\rm M}}_{{\rm{priors}}})$ values across participants among the experiments, priors, and trial bins (*R*^*2*^ = 0.91) (see Supplementary Fig. 2). Thus, the ${\bar{T}}_{R}({{\rm M}}_{{\rm{priors}}})$ values across participants can be used for explaining the ${\hat{\mu }}_{{\rm{prior}}}$ values.

In theory, ${\hat{\mu }}_{{\rm{prior}}}$ can be expressed as a function of ${\bar{T}}_{R}({{\rm M}}_{{\rm{priors}}})$ as follows:

5 $${\hat{\mu }}_{{\rm{prior}}}=\frac{{w}^{2}{[1059\,{ms}]}^{2}{+\sigma }_{\text{prior}}^{2}}{{w}^{2}{[1059\,{ms}]}^{2}}{\bar{T}}_{\text{R}}\left({{\rm M}}_{{\rm{priors}}}\right)-\frac{{\sigma }_{\text{prior}}^{2}}{{w}^{2}[1059\,{ms}]}$$

which can be obtained by resolving Eq. 4 after substituting 1059 ms and ${\bar{T}}_{R}({{\rm {M}}}_{{\rm{priors}}})$ into *T*_*S*_ and ${\bar{T}}_{R}$, respectively. Assuming that *σ*_prior_ and *w* are constant, Eq. 5 is a linear function of ${\bar{T}}_{R}({{\rm M}}_{{\rm{priors}}})$. The significant correlation between the ${\hat{\mu }}_{{\rm{prior}}}$ and ${\bar{T}}_{R}({{\rm M}}_{{\rm{priors}}})$ values (Supplementary Fig. 2) might imply that there was no difference in the *σ*_prior_ and *w* values among the experiments, priors, and trial bins. Alternatively, although there might be a difference in *σ*_prior_ or *w* among them, the effects would be relatively too small to affect the linear correlation between the ${\hat{\mu }}_{{\rm{prior}}}$ and ${\bar{T}}_{R}({{\rm M}}_{{\rm{priors}}})$ values.

In addition, we also evaluated the degree of the central tendency for the short and long priors in Experiments 1–5 and Preliminary Experiment II using the regression index^15,16^, which was calculated by subtracting the slope of the regression line of ${\bar{T}}_{R}$ against *T*_*S*_ from one (1 – slope). As shown in the results of Preliminary Experiment I (Fig. 2c), the ${\bar{T}}_{R}$ × *T*_*S*_ function should be essentially modelled by the nonlinear function as Eq. 4 in the current experiments. However, the linear slope has been widely used for evaluating the central tendency in previous studies, which were well fitted with the results^5,15–17^. There should be no practical problem in approximating the ${\bar{T}}_{R}$ × *T*_*S*_ function using the linear function as in Eq. 2 when calculating within short ranges of *T*_*S*_ as done in previous studies (e.g., max – min ≈ 0.5 s). Actually, the Akaike information criterion (AIC)^42^ values were smaller (i.e., indicating fittings were better) when using Eq. 4 for fittings than when using Eq. 2 in Preliminary Experiment I (for details, see Analyses in Supplementary Methods). Meanwhile, the AIC values were not significantly different between when using Eq. 4 and when using Eq. 2 in Preliminary Experiment II.

If participants concurrently learned the two independent priors, the regression indices should be greater than zero for both short and long priors. In addition, assuming the effect of scalar variability across the priors (i.e., *σ*_sensed_ was constant within each prior but was greater in the long prior than in the short prior), the regression index should be greater for the long prior than for short prior. We tested them per 160 trials (80 trials/prior) in each experiment. Notably, the criterion of the regression index >0 is not always a necessary condition to verify that participants learned the prior when ${\hat{\mu }}_{{\rm{prior}}}$ is largely biased to the other prior (see Results of Experiment 3).

The normality for the residuals of the regression indices over Experiments 1–5 was rejected (*p* = 0.0084, Shapiro–Wilk normality test). In each experiment, the normality for the residuals was rejected in Experiments 2 (*p* = 0.0023) and 3 (*p* = 0.013), marginally not rejected in Experiments 1 (*p* = 0.086) and 4 (*p* = 0.054), and not rejected in Experiment 5 (*p* = 0.38). Although the normality was partially not rejected, we conducted one-tailed Wilcoxon signed-rank tests using the exact method on the regression indices in all experiments for strict and consistent statistical evaluations of the indices over the experiments. We calculated the *p*-values using the exact method because the sample size was not large in each experiment (*n* = 8). The exact method does not compute *z*-statistics. Accordingly, we could not compute Pearson’s *r* (= z/$\sqrt{n}$) as the size effect index and showed only *p*-values in the Wilcoxon signed-rank tests. The *p*-values were corrected in each experiment using the Holm method. The Wilcoxon signed-rank tests were carried out using Matlab R2022b with the Statistics and Machine Learning Toolbox.

It may be also notable that there was no trial-to-trail sequential correlation in timing errors (*T*_*R*_ – *T*_*S*_) of the current coincidence timing task (for details, see Supplementary Results and Supplementary Tables 7–9). In contrast, sequential correlations were found in timing errors of rhythm tapping tasks^43^, which was interpreted as a reflection of a sort of memory process from prior responses to subsequent ones^44^. Thus, such a response memory effect did not affect the current results.

### Reporting summary

Further information on research design is available in the Nature Research Reporting Summary linked to this article.

### Supplementary information

Supplemetary Information

Reporting summary

## Data Availability

The datasets generated and/or analysed during the current study are available upon request by contacting the corresponding author (M.M.).

## Code availability

The underlying codes for this study are available upon request by contacting the corresponding author (M.M.).
