# Supplementary material for: Body-part specificity for learning of multiple prior distributions in human coincidence timing
Source: NPJ Sci Learn. 2024 May 2;9:34. doi: 10.1038/s41539-024-00241-x (PMC11066023; doi:10.1038/s41539-024-00241-x)
Supplement: Supplementary file 1 — Supplemetary Information [file 41539_2024_241_MOESM1_ESM.pdf]

Supplementary Information for

Body-part specificity for learning of multiple prior distributions in human coincidence timing

Yoshiki Matsumura[1], Neil W. Roach[2], James Heron[3], Makoto Miyazaki[1,4*]

[1] Graduate School of Integrated Science and Technology, Shizuoka University, Hamamatsu, Japan

[2] School of Psychology, University of Nottingham, Nottingham, United Kingdom

[3] School of Optometry and Vision Science, University of Bradford, Bradford, United Kingdom

[4] Faculty of Informatics, Shizuoka University, Hamamatsu, Japan

*Corresponding author

Email: miyazaki-makoto@inf.shizuoka.ac.jp (M.M.)

This PDF file includes:

Supplementary Methods

Supplementary Results

Supplementary Figures 1 and 2

Supplementary Tables 1 to 9

Supplementary References

Supplementary Methods

Preliminary Experiments I and II

Participants

Sixteen healthy individuals participated in Preliminary Experiments I and II. Eight individuals participated in one of the two preliminary experiments. There was no overlap of participants among Experiments 1–5 and Preliminary Experiments I and II (see Supplementary Table 1 for the profiles of the participants).

Supplementary Table 1. Profiles of the participants

Exp. #	*n*	Female/Male	Left/Right-hande	Age [mean ± SD (min–max)], years
1	8	1/7	1/7	20.8 ± 1.3 (18–22)
2	8	3/5	1/7	22.1 ± 2.6 (19–26)
3	8	2/6	0/8	22.0 ± 1.7 (20–25)
4	8	2/6	2/6	21.4 ± 1.6 (19–24)
5	8	3/5	2/6	21.1 ± 2.4 (19–26)
Pre-I	8	1/7	1/7	21.5 ± 1.1 (20–23)
Pre-II	8	3/4	1/7	20.8 ± 1.4 (18–23)

Pre-I, Preliminary Experiment I; Pre-II, Preliminary Experiment II; SD, standard deviation

Stimuli, Task, and Procedure

The stimuli, task, and procedure in Preliminary Experiments I and II were the same as those in Experiments 1–4 except for the following points. In Preliminary Experiment I, T_S for both the right and left stimuli were randomly sampled from the wide prior distribution

created by combining the short and long priors (424, 565, 706, 847, 988, 1129, 1271, 1412, 1553, and 1694 ms; upper part in Fig. 2c in the main manuscript) during all sessions. In Preliminary Experiment II, T_S for both the right and left stimuli were randomly sampled from either the short or long prior distributions during half of the sessions and another prior during the other half (upper part in Fig. 2d in the main manuscript). The participants conducted the coincidence timing task by pressing a key using only the dominant index finger in Preliminary Experiments I and II as in Experiment 1.

Analyses

There were no differences in $\bar{T}_R$ values across participants between the right and left stimuli in both Preliminary Experiments I and II. The differences in $\bar{T}_R$ (right – left) across participants, trial bins, and T_S were –1.65 (± 45.9) ms for the mean (± standard deviation) or –5.11 (–27.9/26.0) ms for the median (first/third quartile, Q1/Q3) in Preliminary Experiment I, and –0.67 (± 45.5) ms or –4.45 (–24.6/20.1) ms in Preliminary Experiment II, which were not significantly different from zero (I: $p = 0.21$, II: $p = 0.20$, two-tailed Wilcoxon signed rank test [I: $p = 0.0032$, II: $p < 0.001$, Shapiro-Wilk normality test]). Additionally, for each T_S for each trail bin, there were no significant differences in $\bar{T}_R$ across participants between the right and left stimuli (I: $ps_{cor} \geq 0.31$, II: $ps_{cor} = 1.0$, two-tailed Wilcoxon signed rank tests with Hom correction), for which the medians (Q1/Q3) of the uncorrected p values were 0.55 (0.30/0.84) and 0.46 (0.31/0.75) in Preliminary Experiments I and II, respectively. Therefore, we averaged the T_R values for the right and left stimuli to calculate the $\bar{T}_R$ values for each T_S. Then we calculated the $\bar{T}_R(M_{\text{priors}})$ and $\hat{\mu}_{\text{prior}}$ values in Preliminary Experiments I and II and regression indices in Preliminary Experiment II.

For the $\bar{T}_R(M_{\text{priors}})$ values, the normalities for the residuals were not rejected in Preliminary Experiments I (p = 0.28, Shapiro-Wilk normality test) and II (p = 0.75). Therefore, we used ANOVAs in Preliminary Experiments I and II and one-tailed paired t-tests with Holm correction in Preliminary Experiment II to evaluate the $\bar{T}_R(M_{\text{priors}})$ values similarly to Experiments 1−5. For the ANOVAs, the equalities of variance for trial bin were rejected in Preliminary Experiment I (p = 0.0022) and marginally not rejected in Preliminary Experiment II (p = 0.056) by Mendoza's multisample sphericity test. Therefore, we adjusted the degrees of freedom for trial bin in the ANOVAs using Greenhouse–Geisser's ε.

For the regression indices in Preliminary Experiment II, the normality for the residuals was not rejected (p = 0.44, Shapiro-Wilk normality test). However, we used one-tailed Wilcoxon signed-rank tests with Holm correction for the consistent evaluations of the regression indices among Preliminary Experiment II and Experiments 1−5.

The $\bar{T}_R(M_{\text{priors}})$ values were obtained by fitting the $\bar{T}_R$ values as a function of T_S using the nonlinear equation of Eq. 4. Meanwhile, the regression indices were obtained by fitting the $\bar{T}_R$ values as a function of T_S using the linear equation of Eq. 2. We compared the goodness of the fittings using Eq. 4 and Eq. 2 by the Akaike information criterion (AIC) [1]. For Preliminary Experiment I, the AIC values were smaller (i.e., fittings were better) when using Eq. 4 (1904.7 [1869.1/1927.1], median [Q1/Q3] across fittings) than when using Eq. 2 (1913.6 [1890.6/1937.2]) (p = 0.019, two-tailed Wilcoxon signed rank test [p = 0.051, Shapiro-Wilk normality test]). Meanwhile, for Preliminary Experiment II, the AIC values were not different between when using Eq. 4 and when using Eq. 2 for both the short prior (Eq. 4: 885.6 [857.4/918.3], Eq. 2: 888.3 [859.1/914.7], p = 0.64, two-tailed Wilcoxon signed rank test [p < 0.001, Shapiro-Wilk normality test]) and long prior (Eq. 4: 973.2 [960.9/1004.5], Eq. 2: 974.6 [960.2/1004.5], p = 0.11, two-tailed Wilcoxon signed rank test [p = 0.0028, Shapiro-Wilk normality test]). Thus, the $\bar{T}_R \times T_S$ function should be essentially

modelled by Eq. 4 including the effect of scalar variability [2,3]. Meanwhile, when calculating within short ranges of T_S such as those used in Preliminary Experiment II, there was no practical problem in approximating the $\bar{T}_R \times T_S$ function using Eq. 2 that did not include the effect of scalar variability.

Supplementary Results

Preliminary Experiment I

In Preliminary Experiment I, $\bar{T}_R(M_{\text{priors}})$ and $\hat{\mu}_{\text{prior}}$ values were plotted higher than 1059 ms (μ_{prior}) in trials 1–160 but gradually approached 1059 ms along the progression of trials (Supplementary Figure 1a, b). A one-way repeated-measures ANOVA of the $\bar{T}_R(M_{\text{priors}})$ values exhibited a significant main effect of trial bin ($p = 0.037$, $F(1.32, 9.21) = 5.45$, $\eta_p^2 = 0.44$). The results suggested that in the early trials, the participants acquired a longer $\hat{\mu}_{\text{prior}}$ than the actual mean of the wide prior (1059 ms), although they gradually learned the actual mean as $\hat{\mu}_{\text{prior}}$.

Preliminary Experiment II

In Preliminary Experiment II, $\bar{T}_R(M_{\text{priors}})$ and $\hat{\mu}_{\text{prior}}$ values differed between the short and long priors from early trials and had little change over trials (Supplementary Figure 1c, d). The $\bar{T}_R(M_{\text{priors}})$ values were significantly greater for the long prior than for the short prior over all trial bins ($ps_{cor} \leq 0.0040$, $ts(7) \geq 3.67$, Cohen's $ds \geq 1.30$, paired t-test with Holm correction), indicating that the participants quickly learned the short and long priors.

In addition, a two-way repeated-measures ANOVA (2 priors × 4 trial bins) of the $\bar{T}_R(M_{\text{priors}})$ values exhibited a significant main effect of prior ($p < 0.001$, $F(1, 7) = 66.99$, $\eta_p^2 = 0.91$) but revealed a non-significant main effect of trial bin ($p = 0.87$, $F(1.61, 11.24) = 0.092$, $\eta_p^2 = 0.013$). Although the interaction between them was marginally non-significant ($p = 0.090$, $F(3, 21) = 2.47$, $\eta_p^2 = 0.26$), the analyses of simple effects for the interaction between

prior and trial bin revealed that while the effects of prior were significant in all trial bins (*ps* ≤ 0.0079, $Fs(1, 7) \geq 13.49$, $\eta_p^2 s \geq 0.66$), those of trial bin were not significant for both priors (*ps* ≥ 0.31, $Fs(3, 21) \leq 1.26$, $\eta_p^2 s \leq 0.15$). These results suggested that the participants generally completed learning the priors in the early trials.

Supplementary Figure 1e shows regression indices for the short and long priors in Preliminary Experiment II. In all trial bins, the indices were greater than zero for both priors ($ps_{cor} \leq 0.031$, Wilcoxon signed-rank test with Holm correction), and greater for the long prior than for the short prior ($ps_{cor} = 0.016$). The results further supported that the participants quickly learned the priors.

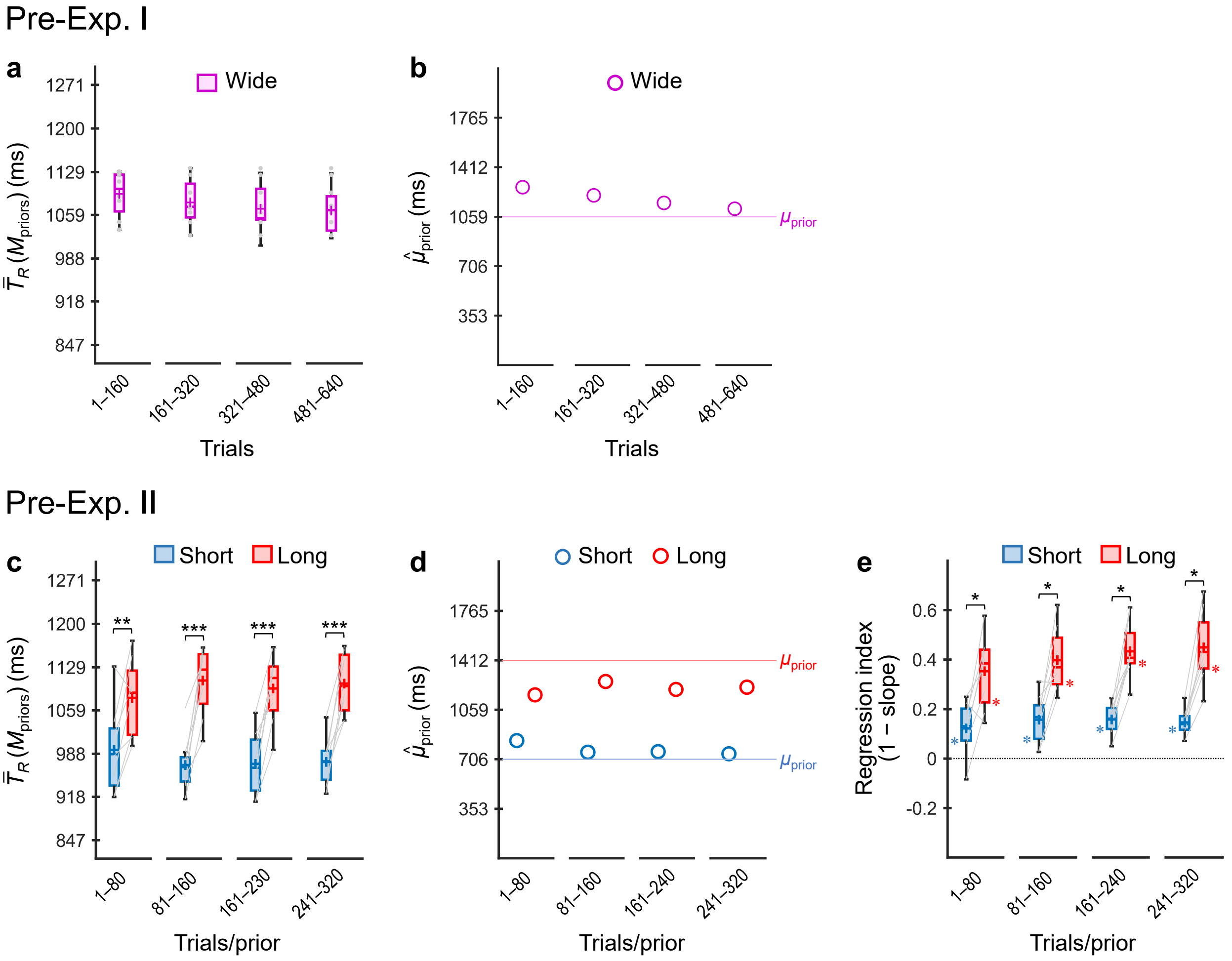

Supplementary Figure 1. Results for Preliminary Experiments I and II. $\bar{T}_R(M_{\text{priors}})$ values across participants for the wide prior in Preliminary Experiment I calculated per 160 trials (**a**). $\hat{\mu}_{\text{prior}}$values inferred using the grand-averaged $\bar{T}_R$ values for the wide prior in Preliminary Experiment I (**b**). $\bar{T}_R(M_{\text{priors}})$ values across participants for the short and long priors in Preliminary Experiment II, calculated per 80 trials/prior (**c**). $\hat{\mu}_{\text{prior}}$values inferred using the grand-averaged $\bar{T}_R$ values for the short and long priors in Preliminary Experiment II (**d**). Regression indices across participants for the short and long priors in Preliminary Experiment II (**e**). The representations of the markers, lines, boxes, and whiskers are the same as those in Figures 4–8 in the main manuscript, except that the $\bar{T}_R(M_{\text{priors}})$ values for each participant were represented as grey dots for Preliminary Experiment I (**a**). * $p_{cor} < 0.05$, ** $p_{cor} < 0.01$, *** $p_{cor} < 0.001$.

Correlation between the $\hat{\mu}_{prior}$ and $\bar{T}_R(M_{priors})$ values

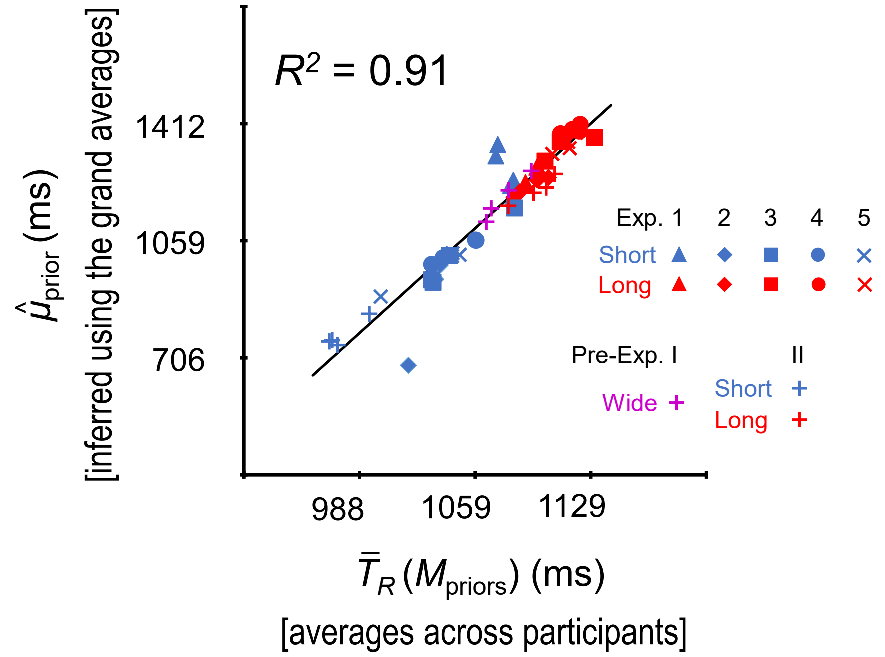

Supplementary Figure 2. Correlation between the $\hat{\mu}_{\text{prior}}$ and $\bar{T}_R(M_{\text{priors}})$ values. Each plot represents the $\hat{\mu}_{\text{prior}}$ and $\bar{T}_R(M_{\text{priors}})$ values calculated for each prior per trial bin in each experiment. The $\hat{\mu}_{\text{prior}}$ values were calculated using the grand-averaged $\bar{T}_R$ values (mean across participants). The $\bar{T}_R(M_{\text{priors}})$ values were calculated for each participant, and then averaged across participants.

Variables obtained by curve fittings in Experiments 1–5 and Preliminary Experiments I and II

Supplementary Tables 2–6 show the $\hat{\mu}_{\text{prior}}$, $\hat{\sigma}_{\text{prior}}$, and w values obtained by the curves fitted to the grand-averaged $\bar{T}_R$ values in Experiments 1–5 and Preliminary Experiments I and II. Bold values in the respective tables were used to account for the discrepancy between the results using the $\bar{T}_R(M_{\text{priors}})$ values and regression indices in trials 1–160 of Experiment 3 (see the last paragraph of 'Experiment 3: timing using two body parts (right vs left hands)' in Results in the main manuscript).

Notably, the $\hat{\mu}_{\text{prior}}$ (μ_{prior} in Eq. 4) is uniquely specified from the fitted curves based on Eq. 4, whereas $\hat{\sigma}_{\text{prior}}$ (σ_{prior} in Eq. 4) and w are unspecified and vary depending on the initial values used for the fittings. To calculate the $\hat{\sigma}_{\text{prior}}$ and w values shown in the tables, two types of initial values were used as follows: (1) $\hat{\mu}_{\text{prior}}$ (short/long) = 1059 ms, $\hat{\sigma}_{\text{prior}}$ (short/long) = 427 ms, w = 0.15 (Supplementary Tables 3 and 4), (2) $\hat{\mu}_{\text{prior}}$ (short) = 706 ms, $\hat{\mu}_{\text{prior}}$ (long) = 1412 ms, $\hat{\sigma}_{\text{prior}}$ (short/long) = 223 ms, w = 0.15 (Supplementary Tables 5 and 6). The initial values of $\hat{\mu}_{\text{prior}}$ and $\hat{\sigma}_{\text{prior}}$ in type (1) were based on the physical statics of the generalised or wide prior; those in type (2) were based on the physical statics of the short and long priors. The initial value of w = 0.15 was commonly used in both types, which was based on a previous study [4].

Supplementary Table 2. The $\hat{\mu}_{\text{prior}}$ values (ms) obtained by fitting the curves based on Eq. 4 to the grand-averaged $\bar{T}_R$ values as a function of T_S

Exp. #	Prior	Trials			
		1–160 (1–80/prior)	161–320 (81–160/prior)	321–480 (161–240/prior)	481–640 (241–320/prior)
1	Short	1226.2	1242.1	1315.0	1349.6
	Long	1234.5	1280.8	1273.8	1225.4
2	Short	1169.5	985.4	942.7	683.4
	Long	1242.3	1249.7	1190.3	1206.6
3	Short	**1158.1**	1014.2	933.9	941.0
	Long	1370.9	1364.0	1358.6	1299.2
4	Short	1060.5	1061.4	1007.7	988.1
	Long	1389.5	1409.6	1382.0	1395.0
5	Short	1021.5	1016.6	1012.7	890.8
	Long	1320.8	1384.5	1356.8	1337.9
Pre-I	Wide	1269.4	1211.2	1156.8	1116.1
Pre-II	Short	838.6	755.6	759.4	744.7
	Long	1164.5	1260.6	1203.5	1219.6

Pre-I, Preliminary Experiment I; Pre-II, Preliminary Experiment II

These $\hat{\mu}_{\text{prior}}$ values were obtained regardless of whether using the initial values (1) or (2), except for the minute difference below significant digits. The bold value (**1158.1** ms) was used to account for the discrepancy between the results using the $\bar{T}_R(M_{\text{priors}})$ values and regression indices in trials 1–160 of Experiment 3.

Supplementary Table 3. The $\hat{\sigma}_{\text{prior}}$ values (ms) obtained by fitting the curves based on Eq. 4 to the grand-averaged $\overline{T}_R$ values as a function of T_S

Exp. #	Prior	Trials			
		**1–160** (1–80/prior)	**161–320** (81–160/prior)	**321–480** (161–240/prior)	**481–640** (241–320/prior)
1	Short	415.8	419.8	460.4	470.8
	Long	382.2	390.4	380.7	398.6
2	Short	381.9	384.8	422.5	505.3
	Long	363.5	378.9	398.3	371.9
3	Short	**365.3**	393.4	367.5	431.1
	Long	360.2	389.2	332.3	390.4
4	Short	334.0	334.2	342.9	336.8
	Long	375.2	381.7	395.8	384.6
5	Short	351.6	358.9	383.0	347.6
	Long	383.9	377.1	372.7	359.9
Pre-I	Wide	378.4	413.7	450.0	457.6
Pre-II	Short	339.8	350.7	341.3	376.3
	Long	397.1	374.5	368.0	360.2

Pre-I, Preliminary Experiment I; Pre-II, Preliminary Experiment II

Type (1) of the initial values were used for fitting. The bold value (**365.3** ms) was used to account for the discrepancy between the results using the $\overline{T}_R(M_{\text{priors}})$ values and regression indices in trials 1–160 of Experiment 3.

Supplementary Table 4. The *w* values obtained by fitting the curves based on Eq. 4 to the grand-averaged $\overline{T}_R$ values as a function of T_S

Exp. #	Prior	Trials			
		**1–160** (1–80/prior)	**161–320** (81–160/prior)	**321–480** (161–240/prior)	**481–640** (241–320/prior)
1	Short	0.157	0.154	0.123	0.118
	Long	0.163	0.163	0.165	0.159
2	Short	0.181	0.185	0.152	0.126
	Long	0.166	0.179	0.174	0.163
3	Short	**0.194**	0.177	0.165	0.149
	Long	0.175	0.166	0.141	0.169
4	Short	0.218	0.219	0.215	0.221
	Long	0.171	0.169	0.163	0.168
5	Short	0.206	0.202	0.186	0.226
	Long	0.166	0.171	0.170	0.173
Pre-I	Wide	0.160	0.154	0.144	0.157
Pre-II	Short	0.203	0.204	0.207	0.210
	Long	0.174	0.194	0.196	0.203

Pre-I, Preliminary Experiment I; Pre-II, Preliminary Experiment II

Type (1) of the initial values were used for fittings. The bold value (**0.194**) was used to account for the discrepancy between the results using the $\overline{T}_R(M_{\text{priors}})$ values and regression indices in trials 1–160 of Experiment 3.

Supplementary Table 5. The $\hat{\sigma}_{\text{prior}}$ values (ms) obtained by fitting the curves based on Eq. 4 to the grand-averaged $\bar{T}_R$ values as a function of T_S

Exp. #	Prior	Trials			
		**1–160 (1–80/prior)**	**161–320 (81–160/prior)**	**321–480 (161–240/prior)**	**481–640 (241–320/prior)**
1	Short	296.6	299.3	381.2	385.6
	Long	341.0	312.8	306.0	304.4
2	Short	270.5	283.8	349.6	436.7
	Long	284.3	279.7	290.1	291.0
3	Short	**256.9**	294.2	300.3	358.4
	Long	284.7	311.3	311.1	257.3
4	Short	226.7	226.7	233.0	226.0
	Long	297.3	303.3	313.1	307.2
5	Short	242.2	248.3	281.3	229.2
	Long	308.6	298.3	298.0	287.2
Pre-I	Wide	313.8	341.5	376.2	360.3
Pre-II	Short	243.4	248.6	240.8	254.2
	Long	288.8	264.9	259.2	251.5

Pre-I, Preliminary Experiment I; Pre-II, Preliminary Experiment II

Type (2) of the initial values were used for fittings. The bold value (**256.9** ms) was used to account for the discrepancy between the results using the $\bar{T}_R(M_{\text{priors}})$ values and regression indices in trials 1–160 of Experiment 3.

Supplementary Table 6. The *w* values obtained by fitting the curves based on Eq. 4 to the grand-averaged $\overline{T}_R$ values as a function of T_S

Exp. #	Prior	Trials			
		**1–160** (1–80/prior)	**161–320** (81–160/prior)	**321–480** (161–240/prior)	**481–640** (241–320/prior)
1	Short	0.112	0.110	0.101	0.097
	Long	0.145	0.130	0.133	0.121
2	Short	0.128	0.136	0.126	0.109
	Long	0.130	0.132	0.126	0.127
3	Short	**0.136**	0.133	0.135	0.124
	Long	0.138	0.133	0.132	0.111
4	Short	0.148	0.148	0.146	0.149
	Long	0.136	0.135	0.129	0.134
5	Short	0.142	0.140	0.137	0.149
	Long	0.133	0.135	0.136	0.138
Pre-I	Wide	0.132	0.127	0.120	0.123
Pre-II	Short	0.145	0.145	0.146	0.142
	Long	0.127	0.137	0.138	0.142

Pre-I, Preliminary Experiment I; Pre-II, Preliminary Experiment II

Type (2) of the initial values were used for fittings. The bold value (**0.136**) was used to account for the discrepancy between the results using the $\overline{T}_R(M_{\text{priors}})$ values and regression indices in trials 1–160 of Experiment 3.

Sequential correlation of motor timing errors

Supplementary Table 7 shows the correlation coefficients (r) between the motor timing errors ($T_R - T_S$) for the trials $i - l$ ($l = 1, 2, 4, 8$) and i, and the p-values for the coefficients (uncorrected for multiple comparisons). In our experiments, each participant completed 16 sessions (40 trials/session) of the coincidence timing task. The task was discontinued per session and the participants took a rest at least for 1 min per one session and 5 min per four sessions. Accordingly, we calculated the r- and p-values for each session in each participant. In the table, each median, Q1, and Q3 shows the value across sessions and participants. Over the experiments and trial lags (l), the r values ranged from −0.08 to 0.01 for the medians, and from −0.18 to 0.14 for the Q1s and Q3s. Thus, the r values distributed around zero. The p-values ranged from 0.44 to 0.57 for the medians, and from 0.18 to 0.84 for the Q1s and Q3s. The percentages of the sessions with $p < 0.05$ ranged from 0.8% to 8.6% (3.9% for the median across all experiments and trial lags). If correcting the p-values for multiple comparisons with the Holm method in each experiment, there was no session with $p_{cor} < 0.05$, and the corrected p-values reached 1.0 in 99.6% of the sessions. Thus, there was no sequential correlation in motor timing errors in the current timing task.

Moreover, we separated the sequences of the motor timing errors into those in trials with the short and long priors for Experiments 1–5 and Preliminary Experiment II. We then calculated the r- and p-values within each prior, in which we found similar results.

Supplementary Table 8 shows the r- and p-values for the sequences with the short prior. The r values ranged from −0.08 to 0.01 for the medians, and from −0.30 to 0.23 for the Q1s and Q3s. The p-values ranged from 0.41 to 0.59 for the medians, and from 0.19 to 0.84 for the Q1s and Q3s. The percentages of the sessions with $p < 0.05$ ranged from 0.8 to 7.0% (3.5% for the median). If correcting the p-values for multiple comparisons in each

experiment, there was no session with $p_{cor} < 0.05$, and the corrected p-values reached 1.0 in 99.6% of the sessions.

Supplementary Table 9 shows the r- and p-values for the sequences with the long prior. The r values ranged from −0.12 to 0.02 for the medians, and from −0.26 to 0.25 for the Q1s and Q3s. The p-values ranged from 0.43 to 0.57 for the medians, and from 0.18 to 0.80 for the Q1s and Q3s. The percentages of the sessions with $p < 0.05$ ranged from 1.6% to 7.8% (4.7% for the median). If correcting the p-values for multiple comparisons in each experiment, there was only one session (i.e., 0.036%) with $p_{cor} < 0.05$ at the fourth session of Experiment 4 with $l = 1$ in one participant, and the corrected p-values reached 1.0 in 99.7% of the sessions.

Supplementary Table 7. Correlation coefficients (*r*) between the motor timing errors for the trials *i* – *l* and *i*, and *p*-values for the coefficients

Exp. #			Trial lag (*l*) 1	2	4	8
1	*r*	Median	−0.02	−0.05	−0.03	−0.01
		Q1/Q3	−0.13/0.05	−0.14/0.06	−0.16/0.08	−0.14/0.12
	p	Median	0.54	0.57	0.44	0.48
		Q1/Q3	0.25/0.84	0.33/0.74	0.20/0.77	0.26/0.74
		%< 0.05	2.3%	1.6%	3.1%	1.6%
2	*r*	Median	−0.02	−0.05	−0.05	−0.02
		Q1/Q3	−0.14/0.09	−0.14/0.05	−0.17/0.07	−0.18/0.12
	p	Median	0.47	0.51	0.44	0.46
		Q1/Q3	0.23/0.78	0.30/0.76	0.18/0.73	0.25/0.77
		%< 0.05	3.9%	5.5%	8.6%	5.5%
3	*r*	Median	−0.02	−0.05	−0.02	−0.05
		Q1/Q3	−0.12/0.07	−0.14/0.04	−0.15/0.07	−0.14/0.08
	p	Median	0.55	0.56	0.56	0.57
		Q1/Q3	0.38/0.78	0.32/0.78	0.27/0.79	0.31/0.74
		%< 0.05	0.8%	3.9%	4.7%	3.1%
4	*r*	Median	0.00	−0.07	−0.02	−0.03
		Q1/Q3	−0.10/0.10	−0.15/0.03	−0.13/0.07	−0.17/0.10
	p	Median	0.53	0.54	0.55	0.48
		Q1/Q3	0.33/0.74	0.28/0.75	0.28/0.76	0.24/0.76
		%< 0.05	3.9%	3.9%	3.1%	7.8%
5	*r*	Median	−0.01	−0.04	−0.03	0.01
		Q1/Q3	−0.14/0.09	−0.13/0.08	−0.14/0.07	−0.09/0.13
	p	Median	0.45	0.54	0.52	0.56
		Q1/Q3	0.21/0.69	0.31/0.79	0.27/0.75	0.24/0.80
		%< 0.05	3.9%	4.7%	2.3%	3.9%
Pre-I	*r*	Median	−0.08	−0.07	−0.02	0.00
		Q1/Q3	−0.17/0.04	−0.15/0.05	−0.12/0.09	−0.11/0.14

	p	Median	.46	.47	.57	.47
		Q1/Q3	0.23/0.70	0.25/0.71	0.28/0.72	0.26/0.78
		%< 0.05	3.9%	3.9%	6.3%	3.9%
Pre-II	*r*	Median	−0.02	−0.04	−0.02	−0.06
		Q1/Q3	−0.13/0.08	−0.15/0.07	−0.14/0.09	−0.17/0.07
	p	Median	0.51	0.47	0.51	0.55
		Q1/Q3	0.24/0.75	0.28/0.76	0.30/0.75	0.24/0.71
		%< 0.05	3.1%	3.9%	3.9%	4.7%

Q1/Q3, first/third quartile; %< 0.05, percentage of the sessions with $p < 0.05$; Pre-I, Preliminary Experiment I; Pre-II, Preliminary Experiment II

Supplementary Table 8. Correlation coefficients (*r*) between the motor timing errors for the trials *i* – *l* and *i*, and *p*-values for the coefficients, selectively calculated for the trials with the short prior

Exp. #			Trial lag (*l*) 1	2	4	8
1	*r*	Median	−0.02	−0.03	−0.08	0.00
		Q1/Q3	−0.16/0.11	−0.19/0.12	−0.21/0.05	−0.18/0.18
	p	Median	0.59	0.53	0.58	0.57
		Q1/Q3	0.33/0.79	0.29/0.79	0.33/0.79	0.30/0.76
		%< 0.05	1.6%	2.3%	2.3%	7.0%
2	*r*	Median	−0.03	−0.06	−0.05	−0.05
		Q1/Q3	−0.17/0.10	−0.18/0.11	−0.25/0.13	−0.23/0.23
	p	Median	0.55	0.52	0.46	0.46
		Q1/Q3	0.32/0.78	0.30/0.76	0.21/0.75	0.21/0.69
		%< 0.05	3.1%	3.9%	5.5%	7.0%
3	*r*	Median	−0.07	−0.05	−0.04	0.01
		Q1/Q3	−0.17/0.09	−0.23/0.16	−0.25/0.12	−0.23/0.14
	p	Median	0.58	0.41	0.48	0.59
		Q1/Q3	0.32/0.76	0.23/0.71	0.25/0.79	0.29/0.82
		%< 0.05	3.1%	6.3%	3.9%	7.0%
4	*r*	Median	0.00	−0.03	−0.05	0.01
		Q1/Q3	−0.16/0.14	−0.19/0.11	−0.20/0.10	−0.17/0.23
	p	Median	0.56	0.51	0.52	0.52
		Q1/Q3	0.33/0.77	0.24/0.84	0.27/0.78	0.30/0.75
		%< 0.05	4.7%	2.3%	3.9%	0.8%
5	*r*	Median	0.00	−0.04	−0.06	−0.05
		Q1/Q3	−0.17/0.15	−0.18/0.10	−0.22/0.12	−0.30/0.16
	p	Median	0.51	0.53	0.56	0.49
		Q1/Q3	0.33/0.75	0.32/0.77	0.28/0.76	0.19/0.77
		%< 0.05	3.1%	3.1%	2.3%	5.5%

Pre-II	*r*	Median	0.00	−0.01	−0.02	−0.05
		Q1/Q3	−0.11/0.08	−0.13/0.09	−0.14/0.08	−0.15/0.07
	p	Median	0.56	0.52	0.58	0.55
		Q1/Q3	0.32/0.74	0.20/0.80	0.30/0.76	0.25/0.74
		%< 0.05	3.1%	1.6%	6.3%	4.7%

Q1/Q3, first/third quartile; %< 0.05, percentage of the sessions with $p < 0.05$; Pre-I, Preliminary Experiment I; Pre-II, Preliminary Experiment II

Supplementary Table 9. Correlation coefficients (*r*) between the motor timing errors for the trials *i* – *l* and *i*, and *p*-values for the coefficients, selectively calculated for the trials with the long prior

			Trial lag (*l*)			
Exp. #			1	2	4	8
1	*r*	Median	−0.09	−0.04	−0.05	−0.09
		Q1/Q3	−0.24/0.07	−0.20/0.11	−0.23/0.09	−0.26/0.09
	p	Median	0.53	0.55	0.50	0.55
		Q1/Q3	0.27/0.74	0.35/0.79	0.29/0.77	0.30/0.77
		%< 0.05	2.3%	3.9%	3.1%	5.5%
2	*r*	Median	−0.06	−0.07	−0.05	−0.03
		Q1/Q3	−0.22/0.07	−0.24/0.11	−0.21/0.12	−0.22/0.19
	p	Median	0.56	0.48	0.50	0.53
		Q1/Q3	0.25/0.78	0.21/0.75	0.27/0.75	0.33/0.75
		%< 0.05	5.5%	3.9%	3.1%	2.3%
3	*r*	Median	−0.12	−0.01	−0.03	−0.01
		Q1/Q3	−0.23/0.02	−0.22/0.14	−0.20/0.16	−0.24/0.25
	p	Median	0.53	0.51	0.52	0.44
		Q1/Q3	0.23/0.72	0.27/0.77	0.25/0.80	0.20/0.70
		%< 0.05	5.5%	3.9%	4.7%	7.8%
4	*r*	Median	−0.08	−0.06	−0.08	−0.05
		Q1/Q3	−0.20/0.07	−0.21/0.16	−0.25/0.08	−0.26/0.22
	p	Median	0.57	0.43	0.56	0.46
		Q1/Q3	0.30/0.77	0.26/0.74	0.24/0.76	0.22/0.69
		%< 0.05	7.0%	7.0%	4.7%	7.0%
5	*r*	Median	−0.10	−0.08	−0.03	0.02
		Q1/Q3	−0.24/0.04	−0.25/0.08	−0.19/0.20	−0.20/0.19
	p	Median	0.49	0.48	0.49	0.55
		Q1/Q3	0.27/0.75	0.26/0.74	0.25/0.69	0.28/0.76
		%< 0.05	4.7%	4.7%	4.7%	3.9%

Pre-II	*r*	Median	−0.04	−0.06	−0.01	−0.08
		Q1/Q3	−0.17/0.05	−0.15/0.05	−0.12/0.12	−0.18/0.07
	p	Median	0.48	0.43	0.47	0.55
		Q1/Q3	0.18/0.77	0.31/0.76	0.31/0.68	0.21/0.68
		%< 0.05	3.1%	6.3%	1.6%	4.7%

Q1/Q3, first/third quartile; %< 0.05, percentage of the sessions with $p < 0.05$; Pre-I, Preliminary Experiment I; Pre-II, Preliminary Experiment II

Supplementary References

1 Akaike, H. A new look at the statistical model identification. *IEEE Trans. Automatic. Control.* **16**, 716-723 (1974).

2 Buhusi, C. V. & Meck, W. H. What makes us tick? Functional and neural mechanisms of interval timing. *Nat. Rev. Neurosci.* **6**, 755-765 (2005).

3 Gibbon, J., Church, R. M. & Meck, W. H. Scalar timing in memory. *Ann. N. Y. Acad. Sci.* **423**, 52-77 (1984).

4 Cicchini, G. M., Arrighi, R., Cecchetti, L., Giusti, M. & Burr, D. C. Optimal encoding of interval timing in expert percussionists. *J. Neurosci.* **32**, 1056-1060 (2012).
