# Supplementary material for: Body-part specificity for learning of multiple prior distributions in human coincidence timing
Source: NPJ Sci Learn. 2024 May 2;9:34. doi: 10.1038/s41539-024-00241-x (PMC11066023; doi:10.1038/s41539-024-00241-x)
Supplement: Supplementary file 2 — Reporting summary [file 41539_2024_241_MOESM2_ESM.pdf]

nature portfolio

Corresponding author(s): Makoto Miyazaki
Last updated by author(s): 12 April, 2024

Reporting Summary

Nature Portfolio wishes to improve the reproducibility of the work that we publish. This form provides structure for consistency and transparency in reporting. For further information on Nature Portfolio policies, see our Editorial Policies and the Editorial Policy Checklist.

Please do not complete any field with "not applicable" or n/a. Refer to the help text for what text to use if an item is not relevant to your study.
For final submission: please carefully check your responses for accuracy; you will not be able to make changes later.

Statistics

For all statistical analyses, confirm that the following items are present in the figure legend, table legend, main text, or Methods section.

n/a	Confirmed	
☐	☒	The exact sample size (*n*) for each experimental group/condition, given as a discrete number and unit of measurement
☐	☒	A statement on whether measurements were taken from distinct samples or whether the same sample was measured repeatedly
☐	☒	The statistical test(s) used AND whether they are one- or two-sided<br>*Only common tests should be described solely by name; describe more complex techniques in the Methods section.*
☐	☒	A description of all covariates tested
☐	☒	A description of any assumptions or corrections, such as tests of normality and adjustment for multiple comparisons
☐	☒	A full description of the statistical parameters including central tendency (e.g. means) or other basic estimates (e.g. regression coefficient) AND variation (e.g. standard deviation) or associated estimates of uncertainty (e.g. confidence intervals)
☐	☒	For null hypothesis testing, the test statistic (e.g. *F*, *t*, *r*) with confidence intervals, effect sizes, degrees of freedom and *P* value noted<br>*Give P values as exact values whenever suitable.*
☒	☐	For Bayesian analysis, information on the choice of priors and Markov chain Monte Carlo settings
☒	☐	For hierarchical and complex designs, identification of the appropriate level for tests and full reporting of outcomes
☐	☒	Estimates of effect sizes (e.g. Cohen's *d*, Pearson's *r*), indicating how they were calculated

Our web collection on statistics for biologists contains articles on many of the points above.

Software and code

Policy information about availability of computer code

Data collection	We used NBS Presentation (Neurobehavioral Systems, USA) for data collection.
Data analysis	We used Matlab R2022b with the Optimization Toolbox and Statistics and Machine Learning Toolbox, R 4.22, Anovakun 4.8.7, and Excel for data analyses.

For manuscripts utilizing custom algorithms or software that are central to the research but not yet described in published literature, software must be made available to editors and reviewers. We strongly encourage code deposition in a community repository (e.g. GitHub). See the Nature Portfolio guidelines for submitting code & software for further information.

Data

Policy information about availability of data

All manuscripts must include a data availability statement. This statement should provide the following information, where applicable:

- Accession codes, unique identifiers, or web links for publicly available datasets
- A description of any restrictions on data availability
- For clinical datasets or third party data, please ensure that the statement adheres to our policy

The datasets generated and/or analysed during the current study are available upon request by contacting the corresponding author (M.M.).

Research involving human participants, their data, or biological material

Policy information about studies with human participants or human data. See also policy information about sex, gender (identity/presentation), and sexual orientation and race, ethnicity and racism.

Reporting on sex and gender	In this study, we recruited participants regardless of their sex and gender.
Reporting on race, ethnicity, or other socially relevant groupings	In this study, we recruited participants regardless of their race, ethnicity, or other socially relevant groupings.
Population characteristics	We recorded the self-reported sex and age of the participants (cf. Supplementary Table 1). The resultant sex ratio (female/male) for each experiment was as follows: 1/7 for Exp. 1, 3/5 for Exp. 2, 2/6 for Exp. 3, 2/6 for Exp. 4, 3/5 for Exp. 5, 1/7 for Pre-Exp I, and 3/4 for Pre-Exp. II. The age [mean (min–max), years] of the participants were 20.8 (18–22) for Exp. 1, 22.1 (19–26) for Exp. 2, 22.0 (20–25) for Exp. 3, 21.4 (19–24) for Exp. 4, 21.1 (19–26) for Exp. 5, 21.5 (20–23) for Pre-Exp. I, and 20.8 (18–23) for Pre-Exp. II.
Recruitment	We recruited healthy individuals as participants by advertising through posters circulated at universities in or around Hamamatsu city.
Ethics oversight	This study was approved by the Ethics Committee of Shizuoka University (15-19). All experiments were performed in accordance with the approved guidelines and regulations. All participants provided written informed consent.

Note that full information on the approval of the study protocol must also be provided in the manuscript.

Field-specific reporting

Please select the one below that is the best fit for your research. If you are not sure, read the appropriate sections before making your selection.

☐ Life sciences ☒ Behavioural & social sciences ☐ Ecological, evolutionary & environmental sciences

For a reference copy of the document with all sections, see nature.com/documents/nr-reporting-summary-flat.pdf

Behavioural & social sciences study design

All studies must disclose on these points even when the disclosure is negative.

Study description	In this study, participants performed a coincidence timing task in which they pressed a key using their right/left index finger, middle finger, or heel according to the timing of visual stimuli. Using the time intervals between the response and stimulus onsets as quantitative data, we assessed whether the participants could learn two prior distributions of target timing (short or long intervals) based on the theoretical predictions of a Bayesian estimation model.
Research sample	A total of 56 healthy individuals participated in a series of seven experiments (Experiments 1–5 and Preliminary Experiments I and II, eight participants per experiment). Each participant performed 640 trials (40 trials/session × 16 sessions) of the timing task.
Sampling strategy	All participants were naïve to the purpose of the experiments. There was no overlap of the participants among the experiments to avoid possible prior learning effects. To minimise the effects of fatigue or drowsiness on task performance, we set a 1-min break after each session and a 5-min break after four sessions. When the participants reported fatigue or drowsiness, the break was extended.
Data collection	We collected participants' responses using a workstation (Precision 3430, DELL, USA) with NBS Presentation (Neurobehavioral Systems, USA).
Timing	Date was collected between 2019 and 2022.
Data exclusions	We excluded trials containing any of the following responses: no key pressing, pressing the opposite key, and pressing the key twice or more. The mean rate of excluded responses was 0.38% over all experiments (Exp. 1: 0.08%, Exp. 2: 0.45%, Exp. 3: 0.51%, Exp. 4: 0.72%, Exp. 5: 0.64%, Pre-Exp. I: 0.08%, Pre-Exp. II: 0.18%).
Non-participation	We excluded any individuals who had any self-reported psychological or neurological disorders or symptoms. We also did not include those who had a poor health condition such as cold symptoms on the day of the experiments. These exclusion criteria were indicated on the recruitment posters. In addition, we screened each applicant using a questionnaire and thermometry immediately before the experiment and did not include those who did not meet the necessary criteria.
Randomization	We randomly recruited healthy individuals as participants. They were recruited from universities in or around Hamamatsu city. We assigned the applicants to the experimental groups in the order in which they applied.

Reporting for specific materials, systems and methods

We require information from authors about some types of materials, experimental systems and methods used in many studies. Here, indicate whether each material, system or method listed is relevant to your study. If you are not sure if a list item applies to your research, read the appropriate section before selecting a response.

Materials & experimental systems

n/a	Involved in the study
☒	☐ Antibodies
☒	☐ Eukaryotic cell lines
☒	☐ Palaeontology and archaeology
☒	☐ Animals and other organisms
☒	☐ Clinical data
☒	☐ Dual use research of concern
☒	☐ Plants

Methods

n/a	Involved in the study
☒	☐ ChIP-seq
☒	☐ Flow cytometry
☒	☐ MRI-based neuroimaging

This checklist template is licensed under a Creative Commons Attribution 4.0 International License, which permits use, sharing, adaptation, distribution and reproduction in any medium or format, as long as you give appropriate credit to the original author(s) and the source, provide a link to the Creative Commons license, and indicate if changes were made. The images or other third party material in this article are included in the article's Creative Commons license, unless indicated otherwise in a credit line to the material. If material is not included in the article's Creative Commons license and your intended use is not permitted by statutory regulation or exceeds the permitted use, you will need to obtain permission directly from the copyright holder. To view a copy of this license, visit http://creativecommons.org/licenses/by/4.0/
